# New insight on the structural features of the cytotoxic auristatins MMAE and MMAF revealed by combined NMR spectroscopy and quantum chemical modelling

**DOI:** 10.1038/s41598-017-15674-1

**Published:** 2017-11-21

**Authors:** Mikael P. Johansson, Hannu Maaheimo, Filip S. Ekholm

**Affiliations:** 1 Department of Chemistry, University of Helsinki, PO Box 55, A. I. Virtasen aukio 1, 00014 Helsinki, Finland; 2 VTT Technical Research Centre of Finland Ltd, PO Box 1000, 02044 VTT, Espoo Finland; 3 Glykos Finland Ltd, Viikinkaari 6, 00790 Helsinki, Finland

## Abstract

Antibody-drug conjugates (ADCs) are emerging as a promising class of selective drug delivery systems in the battle against cancer and other diseases. The auristatins monomethyl auristatin E (MMAE) and monomethyl auristatin F (MMAF) appear as the cytotoxic drug in almost half of the *state-of-the-art* ADCs on the market or in late stage clinical trials. Here, we present the first complete NMR spectroscopic characterisation of these challenging molecules, and investigate their structural properties by a combined NMR and quantum chemical modelling approach. We find that in solution, half of the drug molecules are locked in an inactive conformation, severely decreasing their efficiency, and potentially increasing the risk of side-effects. Furthermore, we identify sites susceptible to future modification, in order to potentially improve the performance of these drugs.

## Introduction

The notion of pharmaceutical “magic bullets”, drugs designed to target diseased cells with high precision and specificity^1–8^, was introduced more than a century ago by Nobel laureate Paul Ehrlich. Despite its mature age, the concept is still a vigorous source of inspiration, as evidenced by, *e*.*g*., the somewhat controversial topic of tailor-made precision medicine^9–12^. The legacy of Ehrlich is also recognised in the active research on antibody-drug conjugates (ADCs).

**Figure 1:**
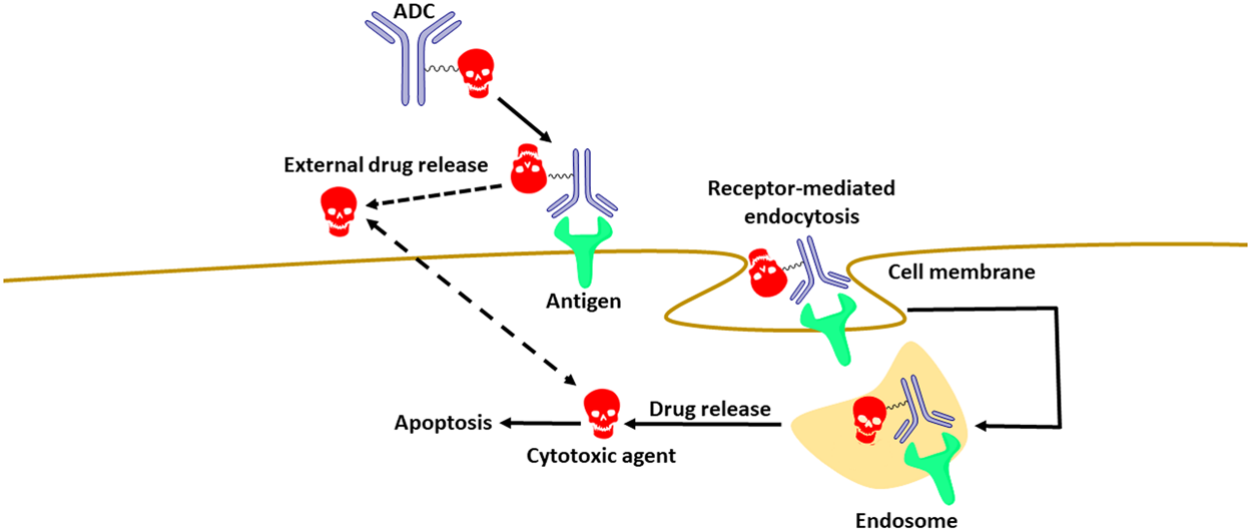
A schematic view of the mode by which ADCs function.

Antibody-drug conjugates are a class of selective drug delivery systems for the treatment of cancers and other diseases^13–18^. The ADCs generally consist of three parts: The antibody (selective targeting unit), the drug, or warhead molecule (a cytotoxic agent), and the chemical linker connecting these. The antibody targets antigens on the diseased cells, after which the entire ADC is internalised by endocytosis. Once inside the cell, the chemical linker is cleaved and the cytotoxic warhead released, thus free to interact with its intended target and induce apoptosis. A schematic view of this pathway is displayed in Fig. 1. Emerging from the days of Ehrlich, through the development of cancer cell specific monoclonal antibodies in the 1970s^19^, the modern ADC era is rich in challenges and diversity, with over fifty ADCs in clinical testing^17,20^. Heterogeneous research teams, combining knowledge in chemistry, biology, and medicine, assemble to deal with the multidisciplinary challenges encountered.

In order to fully understand how the ADCs work, detailed analytical data on the warhead molecules is vital; all of the biological and medical data obtained is entwined with the character of the chemical structures. Standard praxis is to use a combination of mass spectrometry, elemental analysis, and NMR (nuclear magnetic resonance) spectroscopy for the structural determination of organic molecules. The NMR spectroscopic characterisation of organic molecules is especially important due to the wide range of structural information that can be obtained with this method.

Here, we focus on the NMR characterisation of two ubiquitous warhead molecules: Monomethyl auristatin E (MMAE) and monomethyl auristatin F (MMAF). MMAE and MMAF are common cytotoxins in clinical trial ADCs; in 2014, 21 out of 47 ADCs utilised these warheads^21^. Despite wide usage, their detailed NMR spectroscopic data and structural features has, surprisingly, not been previously reported. Access to this information is important not only for determination of their solution structure, but creates novel opportunities for NMR-based molecular recognition studies. Below, we present an accurate NMR spectroscopic analysis, combined with quantum chemical molecular modelling of the auristatins. We find that half of the “magic bullets” fired are actually blanks, that is, inactive in solution. This severely decreases the efficiency of the ADCs, and increases the risk of side-effects. By understanding the underlying reasons, *via* a proper structural characterisation, improved drugs can be designed.

## Results and Discussion

As mentioned, in the majority of the ADCs, the cytotoxic warhead molecule is connected to the antibody by a cleavable chemical linker, which leads to liberation of the cytotoxic agent upon reaching the desired destination. Therefore, understanding the structural properties and dynamic binding mode of the cytotoxic drug is essential for the design of efficient ADCs.

The auristatins MMAE and MMAF are notorious for their complex NMR spectra, to a large extent a result of conformational isomerism due to a partially hindered rotation around the dolaproine-dolaisoleuine amide bond. The complete NMR spectroscopic characterisation of these warhead molecules presented below is thus an important milestone and pre-requisite on the path to study their properties in solution. The excellent NMR spectroscopic data previously reported for naturally occurring dolastatin 10 by Alattia *et al*.^22^ and Benedetti *et al*.^23^ was utilised as a starting point for our current work. Below, we separately discuss the NMR spectroscopic assignments and conformational properties of MMAE and MMAF.

### Structural characterisation and conformational analysis of MMAE

**Figure 2:**
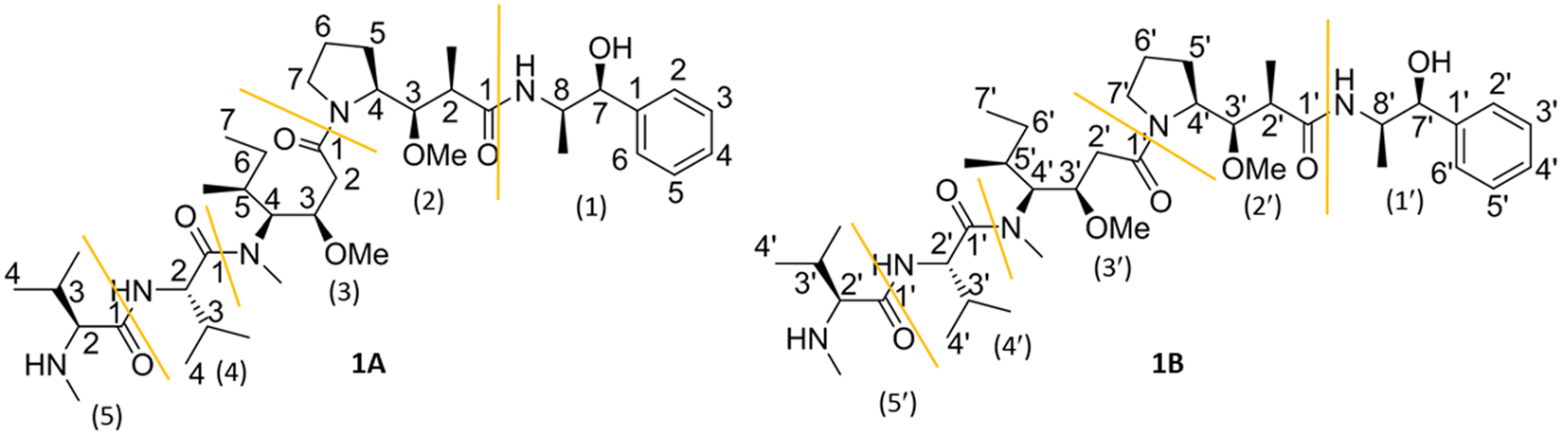
The two conformers of MMAE are distinguished by **1A** (*cis*-conformer) and **1B** (*trans*-conformer). The numbering of the peptide residues utilises parentheses and the numbering of positions conventional numbers. The signals and residues in **1B** are marked with a prime. The peptide residues in MMAE are: (1) norephedrine, (2) dolaproine, (3) dolaisoleuine, (4) valine and (5) monomethyl valine.

The antineoplastic and antimitotic drug MMAE appears as the cytotoxic payload molecule in at least sixteen ADCs which have progressed to clinical trials^21^. Among these is the ADC Brentuximab vedotin which is utilised in the treatment of relapsed cases of Hodgkin’s lymphoma and anaplastic large cell lymphoma^24^. MMAE is composed of five peptide residues and has been reported to exist in solution as a mixture of two conformers due to a partially hindered rotation around the dolaproine-dolaisoleuine amide bond. The chemical structure of the *cis*/*trans*-isomers, information on the amino acid residues and the numbering that will be utilised in the discussion is summarised in Fig. 2.

**Figure 3:**
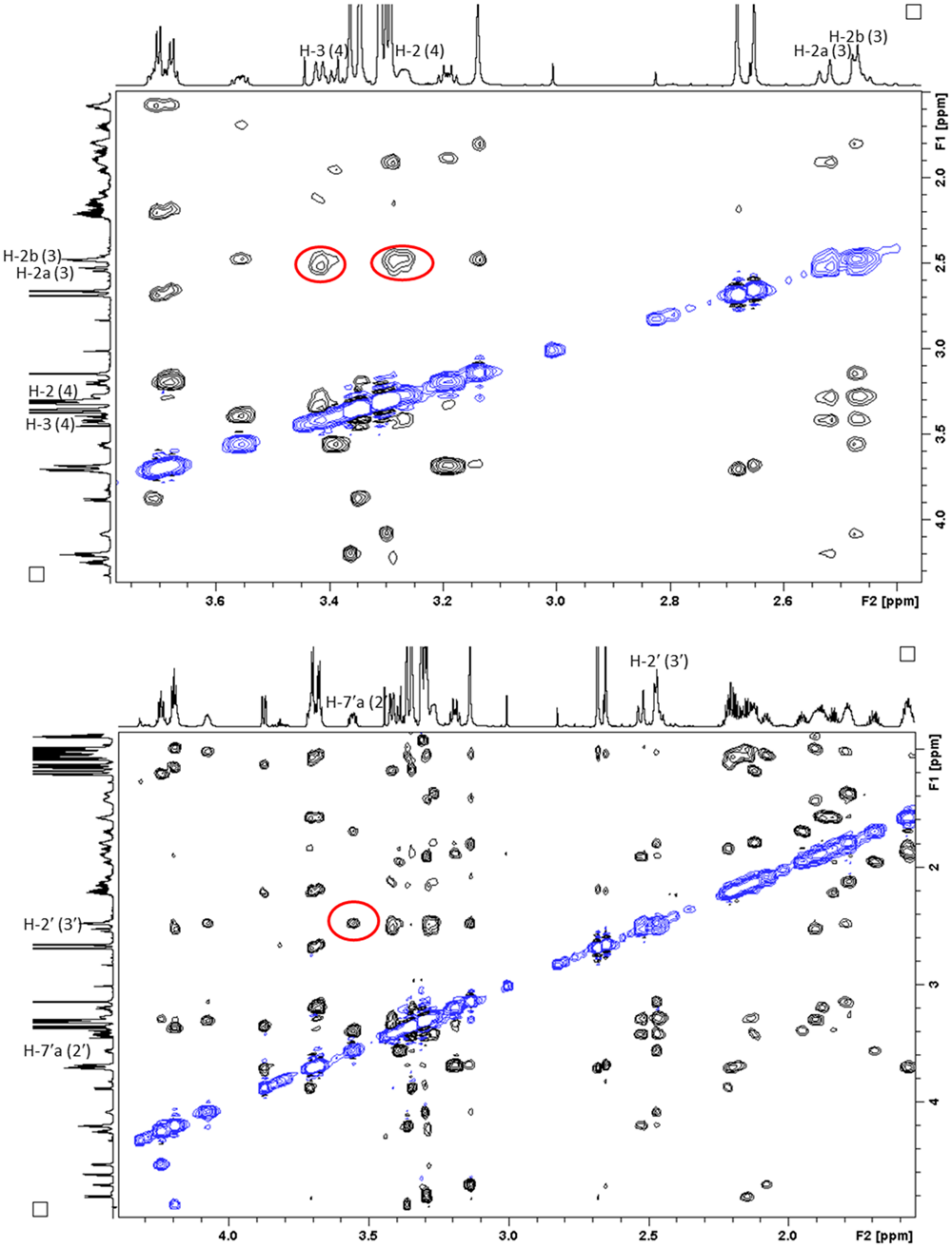
Selected regions of the 2D ROESY spectrum utilised in the determination of the stereoisomers of MMAE. Top: the most important ROE-correlations in residues (2) and (3) of **1A** are highlighted with red circles; bottom: the most important ROE-correlations in residues (2′) and (3′) of **1B** are highlighted with a red circle.

While the appearance of the two conformers of MMAE have been noted before^22,23,25^, reports on the correlation between the individual NMR signals and the molecule are absent in the literature. This was therefore the logical place to start our current work. In order to assign the individual signals in the complex NMR spectra of MMAE, a combination of NMR methods was required. In this study, we utilised an 850 MHz NMR instrument and the following set of NMR spectroscopic techniques: 1D^1^H and ^13^C; 2D COSY (correlation spectroscopy), 2D^13^C multiplicity edited HSQC (heteronuclear single-quantum coherence, edHSQC,), TOCSY (total correlation spectroscopy, both 1D and 2D), 2D HSQC-TOCSY, 2D HMBC (heteronuclear multiple bond correlation) and 2D ROESY (rotating-frame nuclear Overhauser effect spectroscopy). Due to the limited aqueous solubility of MMAE we used deuterated methanol as a solvent. Compared to the popular solvents DMSO-d6 and CD_2_Cl_2_, methanol better represents an aqueous environment, as it is a polar protic solvent which can participate in hydrogen bonding. In fact, Benedetti *et al*. concluded that the conformational properties observed for this class of compounds is solvent dependent, with a significant difference noted between CD_2_Cl_2_ and CD_3_OD^23^. The key methods for identifying and assigning the signals in the complex^1^H- and^13^C-NMR spectra of **1A** and **1B** were high-resolution HMBC and edHSQC (See Figs 3 and 6 in the Supplementary information). COSY, TOCSY (2D) and HSQC-TOCSY were at all stages utilised to verify that the signal assignments were logical and correct. This approach was found to be solid also during the assignment of crowded areas of the spectra. A detailed guide on the NMR spectroscopic characterisation of MMAE is provided in the Supplementary information. The chemical shifts, coupling constants, HMBC correlations and ROE (rotating-frame nuclear Overhauser effect) correlations observed for MMAE are summarised in Tables 1 and 2 in the Supplementary information.

With the NMR spectroscopic characterisation of MMAE completed, the pre-requisites for a more thorough investigation on the conformational properties of conformers **1A** and **1B** was possible. We initiated the conformational characterisation by analysing which of the structures **1A** and **1B** corresponded to the *cis*/*trans*-conformers of MMAE. At the outset of the investigation, the focus was placed on the ROEs observed in the dolaproine and dolaisoleuine residues (2), (2′), (3) and (3′). The ROE correlations observed between H-3 (2) and H-2a (3) and H-4 (2) and H-2b (3) confirmed that **1A** was the *cis*-conformer. On a related note, the ROE correlation between H-7′a (2′) and H-2′ (3′) in **1B** proved that this was the *trans*-conformer (2D ROESY spectrum displayed in Fig. 3).

Examining the chemical shifts of these two residues revealed major differences between the two isomers. The chemical shift difference was found to be greatest for position 3 (H-3 (2) at 3.42 ppm, C-3 (2) at 86.6 ppm) and 3′ (H-3′ (2′) at 3.87 ppm, C-3′ (2′) at 83.5 ppm). The deviation for H-4 (2) and H-4′ (2′) was identical, *i*.*e*., 0.44 ppm; the corresponding carbon signals C-4 (2) and C-4′ (2′) appeared at a similar frequency, however. The H-7′a (2′) and H-7′b (2′) signals were separated by 0.17 ppm while the H-7a (2) and H-7b (2) were separated by 0.49 ppm. While chemical shifts obtained in different solvents cannot be directly compared, the same chemical shift trends observed for MMAE in our work, could also be identified in the NMR spectroscopic data reported for dolastatin 10 in both CD_2_Cl_2_ and DMSO-d6^22,23^. In addition, the H-2 (3)-protons appear to be asymmetric, and appeared as a doublet (d) and doublet of doublets (dd) at different chemical shifts (H-2a (3) as a d at 2.53 ppm, H-2b (3) as a dd at 2.46 ppm) while the H-2′ (3′)-protons appeared as a single d at 2.47 ppm. The differences observed in the chemical shift for the dolaproine-dolaisoleuine signals suggest that the *cis*/*trans*-isomerism has a substantial effect on the three-dimensional structure of MMAE. In addition to the chemical shift deviations observed, the signals in the *cis*-dolaproine residue were much broader than the corresponding signals in the *trans*-dolaproine residue thereby potentially indicating a more complex conformational equilibrium than the one previously assumed. In order to investigate the conformations of the *cis*/*trans*-isomers in more detail, more information on the 3D properties was required.

*A priori*, it would seem a fair assumption that the isomers of MMAE would resemble the structures reported for dolastatin 10^22,23^. However, comparison of the chemical shifts reported for dolastatin 10 and those observed for MMAE in this work revealed a major discrepancy which could not be ignored. The H-2 (5) proton appeared at 2.65 and 2.39 ppm in dolastatin 10 and at 3.70 and 3.68 ppm in MMAE. This indicated that the 3D structure of MMAE is distinct from the one reported for dolastatin 10, although with some of the structural features shared. As a result, a careful and systematic analysis of the 2D-ROESY spectrum of MMAE was performed.

**Figure 4:**
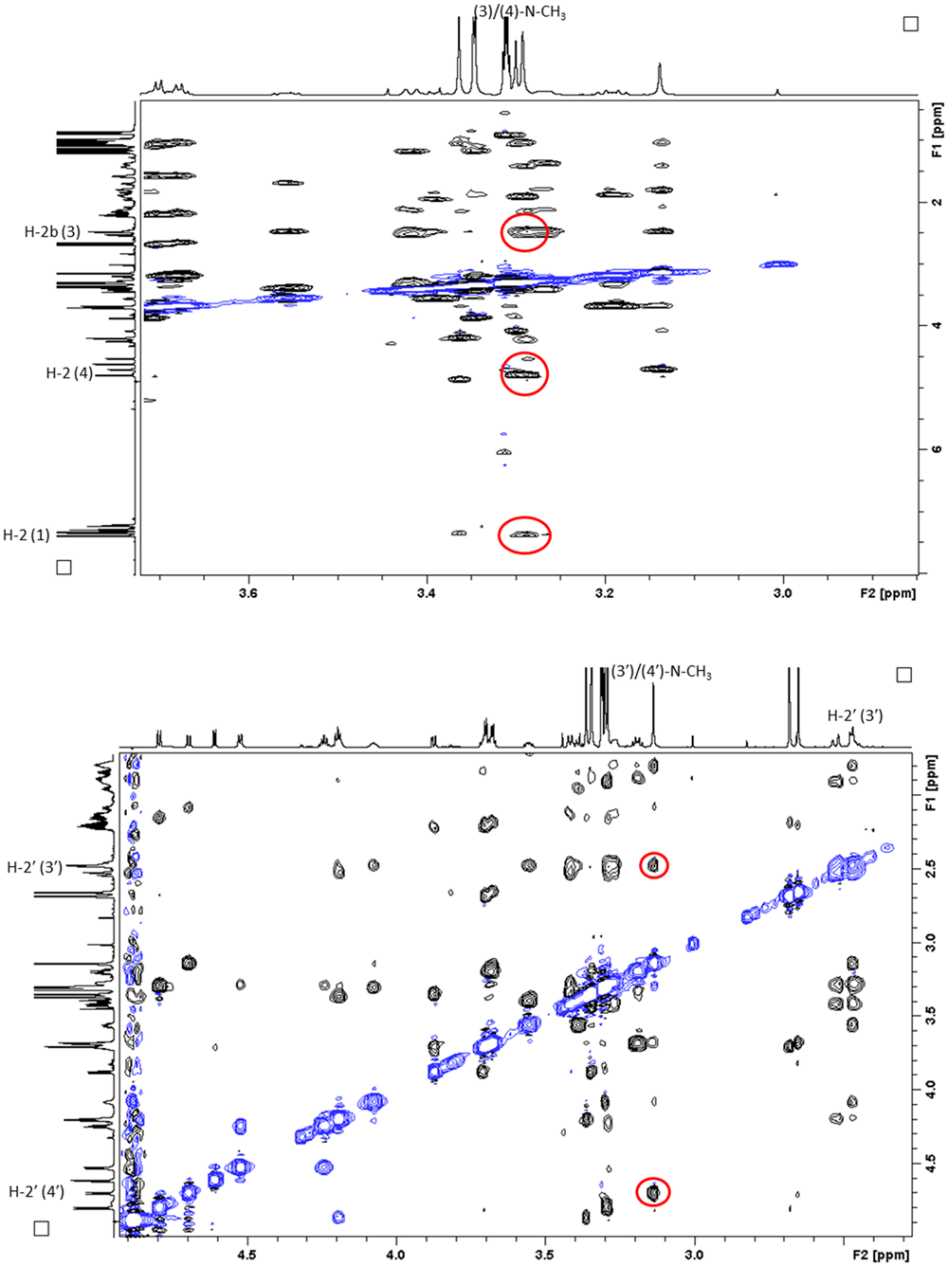
The figures display the key ROE correlations witnessed for **1A** (*cis*-conformer) and **1B** (*trans*-conformer). Top: the 2D ROESY spectrum highlighting the most important ROE correlations observed in **1A** (red circles); bottom: the 2D ROESY spectrum highlighting the most important ROE correlations observed in **1B** (red circles).

Most of the ROEs observed in the spectra of **1A** and **1B** did not offer any information on the three-dimensional features of the isomers, and were therefore of limited assistance. We did, however, discover an unexpected long-range correlation between the aromatic ring and the (3)/(4)-N-CH_3_ in **1A**. This correlation revealed that these non-contiguous parts of the molecule are spatially adjacent. We note that the corresponding correlation has not been observed or reported previously for either auristatins or dolastatins. In addition to this important cross peak, the spatial arrangement of the remaining parts of the molecule could be uncovered based on the other ROE correlations observed for the (3)/(4)-N-CH_3_ group, *i*.*e*., the correlations to both H-2b (3) and H-2 (4). The most important ROE cross peaks are displayed in Fig. 4. The ROE correlations of the *trans*-isomer **1B** were analysed in a similar fashion. Even if no correlations between non-contiguous residues could be observed, the correlation observed between (3′)/(4′)-N-CH_3_ group and H-2′ (4′) and H-2′ (3′) could be utilised to gain insights on the structural features of **1B**. The most important ROE cross peaks of **1B** are summarised in Fig. 4.

**Figure 5:**
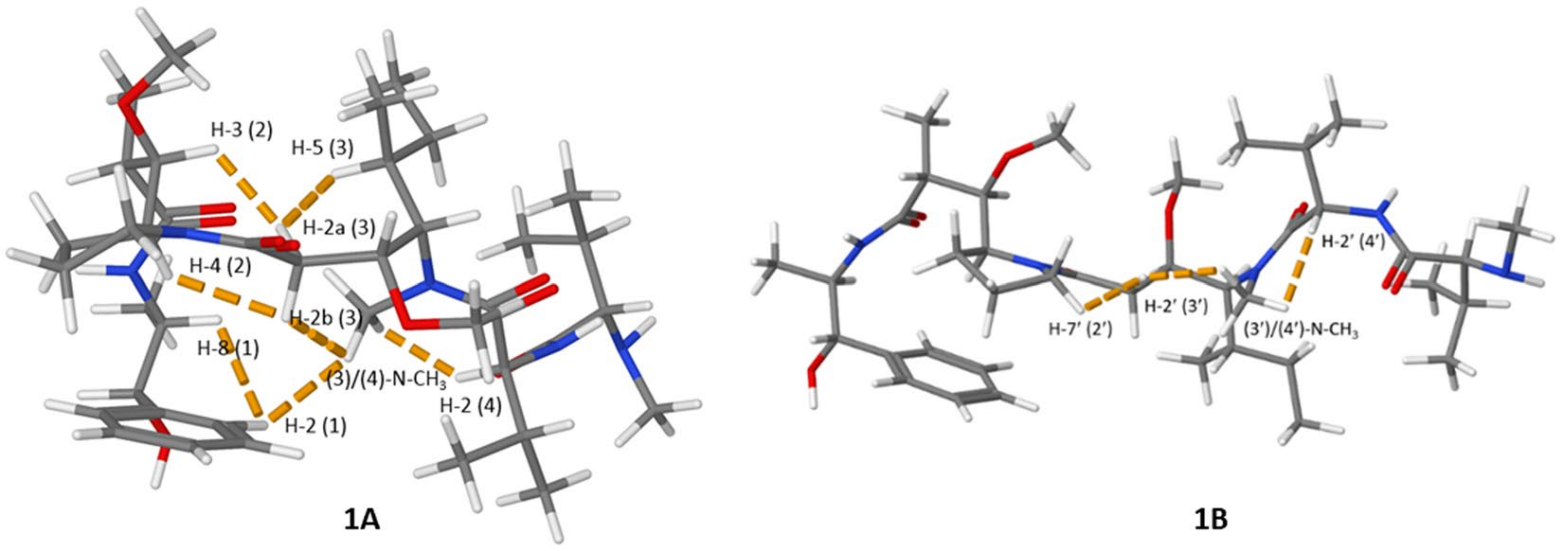
The figures display the modelled low-energy conformations of MMAE; **1A** (*cis*-conformer) and **1B** (*trans*-conformer). The most important experimental ROEs are displayed with dashed orange lines.

The experimental evidence obtained on the solution features of these isomers provided a solid foundation for quantum chemical molecular modelling. The initial molecular models were constructed based on the experimental information uncovered during the NMR-spectroscopic investigation (coupling constants and ROEs). These structures were then freely optimised at density functional theory (DFT) level, while final electronic energies were obtained at spin-component scaled 2^nd^ order perturbation theory level (SCS-MP2)^26^, see Methods. The optimised molecular structures are displayed in Fig. 5 along with the most important ROEs observed. A comparison of the Gibbs free energies for the modelled structures **1A** and **1B** reveal that the *trans*-conformer is marginally less stable than the *cis*-conformer, with ΔG(295 K) = 0.6 kJ/mol, corresponding to a **1A**:**1B** ratio of 56:44. Integration and comparison of the intensities of the NMR signals (measured at 295 K) H-7 (1) vs. H-7′ (1′), 8-CH_3_ (1) vs. 8′-CH_3_ (1′) and 2-CH_3_ (2) vs. 2′-CH_3_ (2′) gave a **1A**:**1B** ratio of 60:40 which is in good agreement with the theoretical calculations. This ratio is also identical to the one previously reported for dolastatin 10 in DMSO-d6^22^. While we conducted the major part of our experimental study at 295 K, we also examined the population distribution at 310 K, a naturally more relevant temperature from a biological standpoint: At 310 K the **1A**:**1B** ratio was found to be 52:48. In addition, despite its poor aqueous solubility, we were able to measure a^1^H-NMR spectrum of MMAE in D_2_O. The population ratios and conformational characteristics were confirmed to be practically identical to those observed in deuterated methanol, with a **1A**:**1B** ratio of 59:41 at 295 K. Examining the molecular models of **1** revealed that the *trans*-conformer, **1B**, forms an “extended” structure which resembles the tubulin-bound form of MMAE^27^. From the experimental data alone, it was not possible to ascertain whether the aromatic ring in the *trans*-conformer would adopt a position under the dolaproine residue, or whether it would turn outwards to form an extended structure. The latter option was, however, ruled out by quantum chemical calculations which show this isomer to be 9 kJ/mol higher in free energy, to a large part due to decreased intramolecular dispersion interaction between the aromatic ring and the rest of the auristatin. The *cis*-conformer **1A**, on the other hand, forms a contorted compact structure which is distinct from the previously reported biologically active conformation. The broader signals observed in the^1^H-NMR spectrum of the dolaproine residue in the *cis*-conformer suggests that this residue is more susceptible to ring puckering than the corresponding residue in the *trans*-conformer.

Our results show that the previously reported bound form of MMAE^27^ is the minor isomer in solution. Based on our calculations, the energy barrier for conversion between these structures is significant, *ca*. 101 kJ/mol, indicating a mean lifetime of several hours at body temperature^28^. Viewed from a biological perspective, these findings suggest that 50–60% of this widely utilised auristatin exists in an inactive conformation with respect to the previously reported binding site.

### Structural characterisation and conformational analysis of MMAF

**Figure 6:**
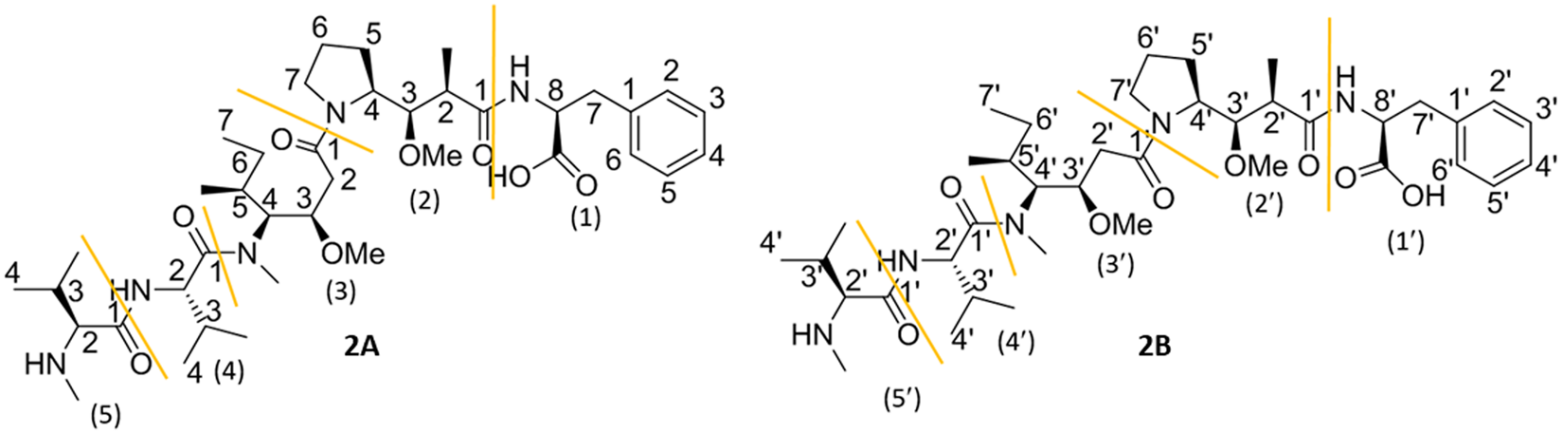
The two isomers of MMAF are distinguished by **2A** (*cis*-conformer) and **2B** (*trans*-conformer). The numbering of the peptide residues utilises parentheses and the numbering of signals conventional numbers. The signals and residues in **2B** are marked with a prime. The peptide residues in MMAF are: (1) phenylalanine, (2) dolaproine, (3) dolaisoleuine, (4) valine and (5) monomethyl valine.

MMAF, also a tubulin polymerase inhibitor, appears as the cytotoxic payload molecule in at least six ADCs which have progressed to clinical trials^21^, and structurally resembles MMAE. In MMAF, the norephedrine residue of MMAE is replaced by phenylalanine. The properties of MMAF are similar to those of MMAE, *i*.*e*., it gives rise to *cis*/*trans*-conformers in solution. A similar numbering system as the one utilised above will be utilised in the discussion on the structural characterisation of MMAF. The chemical structure and numbering of MMAF is summarised in Fig. 6. The NMR spectroscopic characterisation of MMAF is discussed in the Supplementary information. The chemical shifts, coupling constants, HMBC correlations and ROEs witnessed are summarised in the Supplementary Tables 3 and 4.

**Figure 7:**
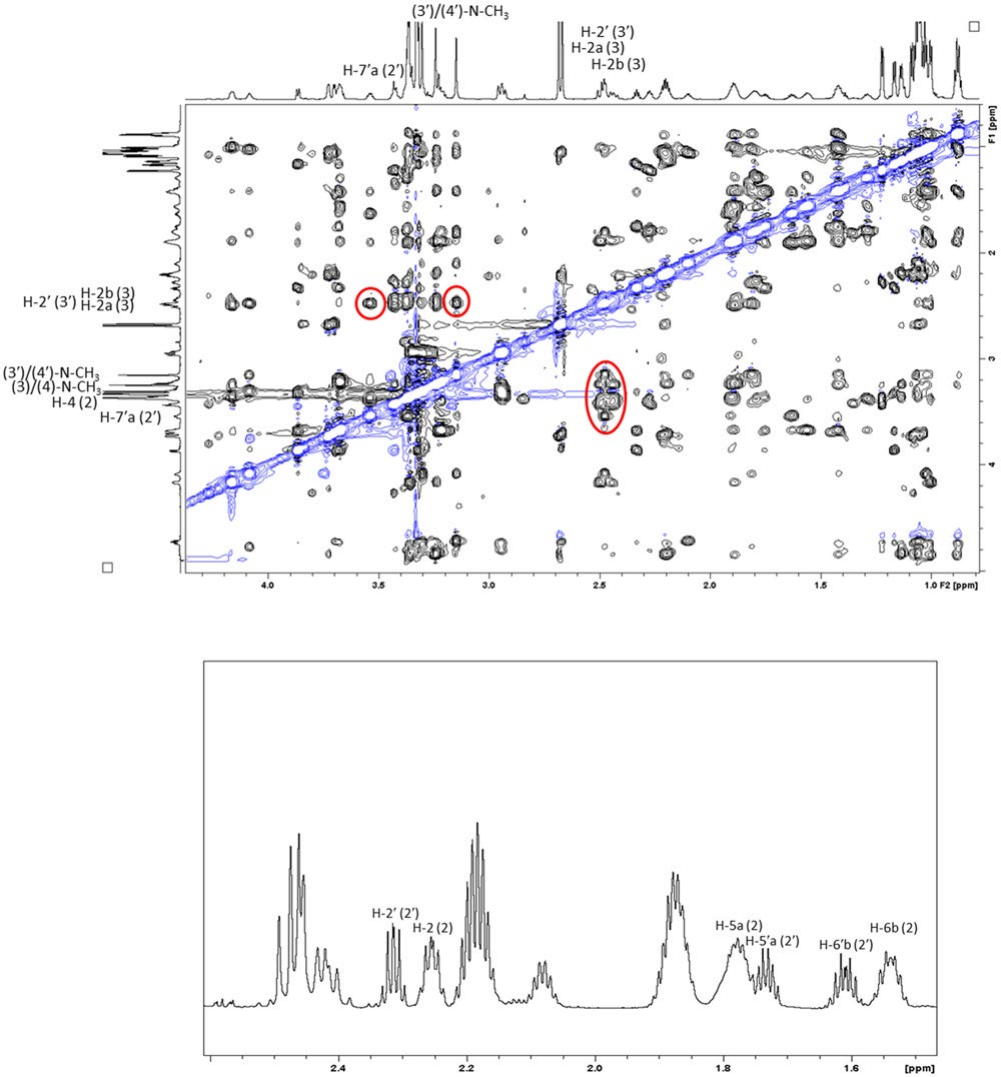
Selected parts of the NMR spectra utilised in the conformational characterisation of MMAF. Top: the 2D ROESY spectrum with the most important cross peaks highlighted with red circles; Bottom: a selected area of the proton spectrum (2.35–1.50 ppm) displaying the large difference in the signal shapes of the two conformers.

With the NMR spectroscopic characterisation of MMAF completed, we continued with the conformational analysis. Again, deuterated methanol was utilized as the primary solvent. The *cis*- and *trans*-conformers could be distinguished based on the ROEs observed in the dolaproine-dolaisoleuine residues (Fig. 7). The most important of these were the ROEs between H-2b (3) and H-4 (2) in the *cis*-conformer (**2A**) and H-7′a (2′) and H-2′ (3′) in the *trans*-conformer (**2B**). The **2A**:**2B** ratio was found to be identical (60:40) to the one observed for MMAE (at 295 K) and dolastatin 10^22,23^. The **2A**:**2B** population ratios were found to be 55:45 at 310 K in deuterated methanol and 55:45 at 295 K in D_2_O. The computed isomerisation free energy (2.2 kJ/mol) implies a Boltzmann ratio of 70:30, again a good agreement between experiment and theory. A careful analysis of the chemical shifts and the signal shapes of the individual signals in MMAF revealed similar trends as the ones reported for MMAE above (especially for residues (2), (2′), (3) and (3′)). The information acquired up to this point suggested that the *cis*- and *trans*-conformers of MMAF bear a structural resemblance to the corresponding isomers of MMAE and are distinct to the ones reported previously for dolastatin 10^22,23^.

**Figure 8:**
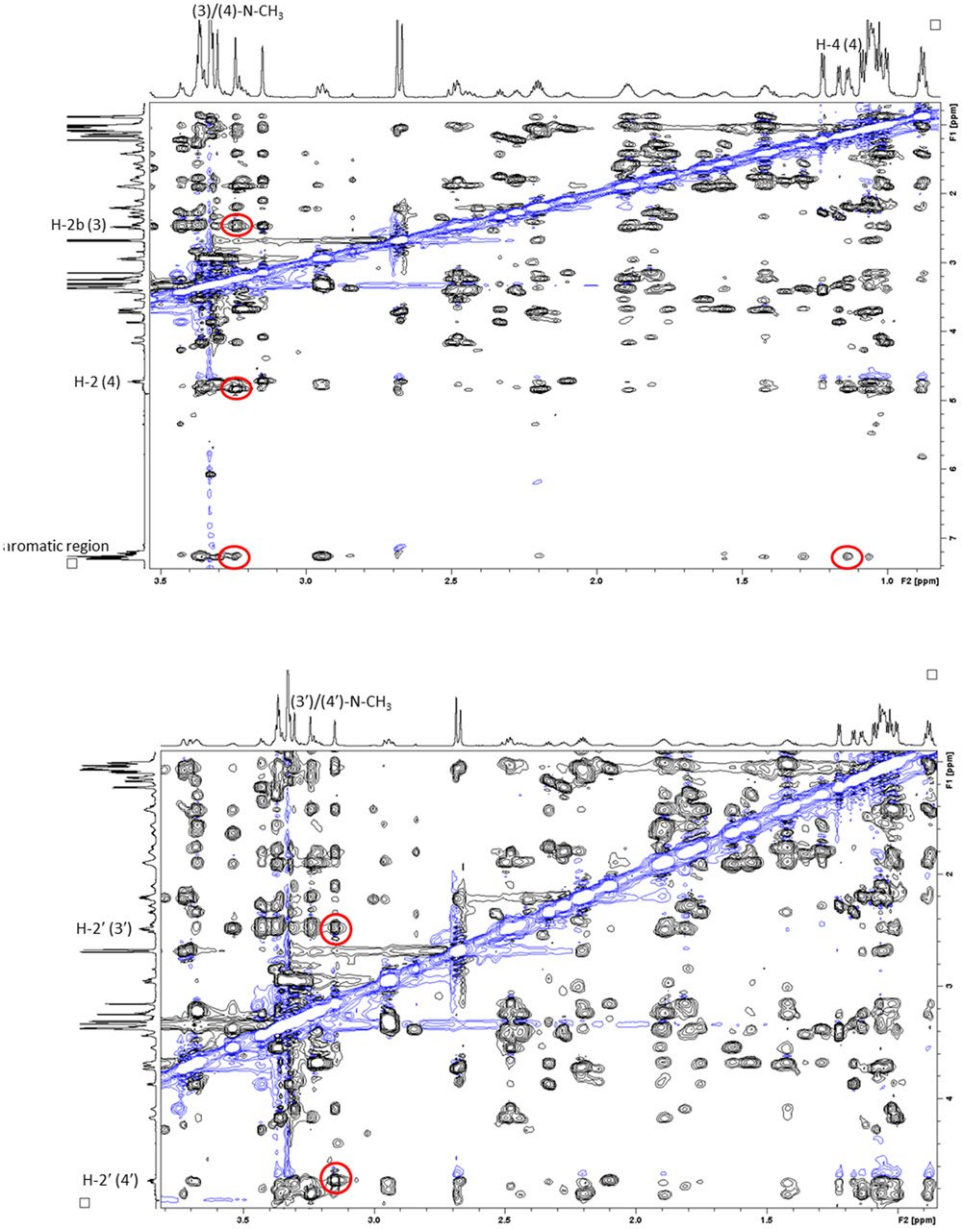
The figures display the key ROE correlations witnessed for **2A** (*cis*-conformer) and **2B** (*trans*-conformer). Top: the 2D ROESY spectrum highlighting the most important ROE correlations observed in **2A** (red circles); bottom: the 2D ROESY spectrum highlighting the most important ROE correlations observed in **2B** (red circles).

A thorough investigation of the 2D ROESY spectrum confirmed that MMAE and MMAF share a number of structural features. In the *cis*-conformer **2A**, the long-range correlation between the aromatic ring and the (3)/(4)-N-CH_3_-group was observed thereby revealing that these non-contiguous residues are spatially adjacent (Fig. 8). Surprisingly, the H-4 (4)-proton was found to be in closer proximity to the aromatic ring than the (3)/(4)-N-CH_3_-group in MMAF (in contrast to MMAE). Apart from this minor deviation, the remaining ROEs were similar in the *cis*-conformers of both auristatins (*i*.*e*., the (3)/(4)-N-CH_3_ correlations to H-2 (4) and H-2b (3) were observed). The net result is that **2A** bears a structural resemblance to **1A**. The ROEs of the *trans*-conformer **2B** were analysed next. As suspected, there were no correlations between non-contiguous residues in **2B**. Nevertheless, the correlations observed between the (3′)/(4′)-N-CH_3_ group and H-2′ (4′) and H-2′ (3′) could be utilised to gain insights on the structural features of **1B** (Fig. 8).

**Figure 9:**
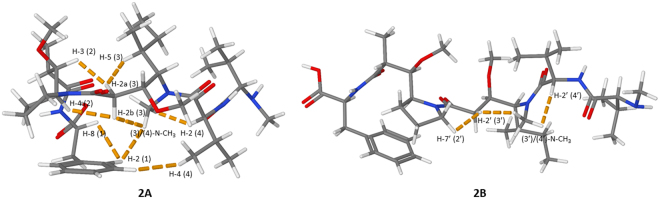
The figures display the modelled low-energy conformations of MMAF; **2A** (*cis*-conformer) and **2B** (*trans*-conformer). The most important experimental ROEs are displayed with dashed orange lines.

The ROEs observed were again utilised in the construction of molecular models of the two conformers of MMAF (Fig. 9). The structural features of the conformers are almost identical to those of MMAE. The *trans*-isomer **2B** forms an extended structure which resembles the previously reported tubulin-bound form^27^, while the *cis*-isomer **2A** forms a contorted compact structure with distinct features. The optimised molecular structures were in good agreement with the experimental results, with, *e*.*g*., the H-4 (4) proton found closer to the aromatic ring than the (3)/(4)-N-CH_3_-group in the *cis*-conformer of MMAF, as also observed in the 2D ROESY spectrum. The broader signals noticed in the *cis*-dolaproine residue indicates that the dolaproine ring is more prone to undergo ring puckering in the *cis*-conformer. Understanding the correlation between these structural features and the biological activity which has been linked to the *trans*-isomer^27^ may be of special interest during the future development of novel and improved auristatins.

We also note that MMAF has the potential to form an intramolecular hydrogen bond involving its carboxylic acid group, which MMAE lacks. Indeed, the simulated conformational search identified an isomer, where two intramolecular hydrogen bonds are present within MMAF, both of N‒H∙∙∙O type (see Supporting Information for coordinates). The computed free energy of this isomer is 15 kJ/mol higher than that of **2A**, and the ROEs observed in the experimental data are not present, so it is ruled out in the present study. Its lack of stability compared to **2A/B** arises largely from a poorer solvent interaction, which destabilises the hydrogen-bonded isomer by 40 kJ/mol compared to **2A**. In a less polar medium, say chloroform with a dielectric constant ε = 4.8, this isomer should, however, come very close in energy to the other isomers.

### Concluding remarks

Structural characterisation data reported for the complex ADC payload molecules is one of the cornerstones that governs the reproducibility and reliability of the scientific results obtained with such substrates and their end products. In terms of ADC research this requires that the data on the synthesis and identity of the payload molecules are accurate and sufficient. Here, we presented a thorough NMR spectroscopic characterisation of the two widely utilised auristatins MMAE and MMAF. The NMR spectroscopic characterisation enabled the study of the three-dimensional features of these auristatins in unprecedented detail, complementing the previous literature data^29–35^.

We found that the major isomer (the *cis*-isomer) observed in solution is distinct from the previously reported tubulin bound form of the auristatins^27,36^. The quantum chemical calculations revealed the energy barrier between the *cis*/*trans*-conformers to be substantial, and their mean lifetime in the hour range^28^. It is uncertain if these hydrophobic cytotoxic agents remain within the target biological surroundings during the course of their mean lifetime. Thus, their medical potential may be significantly diminished and, perhaps even more urgent, the risk for unintended side effects increased.

With this knowledge on the conformational properties of MMAE and MMAF, it is possible to improve their cytotoxic performance by rationally designing appropriate chemical modifications of their molecular structures. These chemical modifications could be aimed at, say, increasing the steric bulk on the molecular entities which are spatially adjacent in the *cis*-conformer, *i*.*e*., the aromatic ring, the (3)/(4)-amino-group and the dolaproine-dolaisoleuine amide bond. Modifications in this region may lead to more favourable isomer ratios, favouring the *trans*-conformer. Another approach would be to decrease the interconversion barrier between the isomers, facilitating activation of the inactive conformation. It would of course also be prudent to perform dynamic binding studies in order to rule out the possibilities of additional binding sites and conformations.

Finally, the NMR data and the conformational analysis reported herein will advance the possibilities to study the dynamic molecular recognition events taking place between the auristatins and their tubulin-receptors by NMR spectroscopy. Such molecular interaction studies have become a robust method for obtaining insights on the recognition events occurring in biological systems^37–40^, and are expected to complement the information obtained from the previously reported ligand-protein co-crystallisation experiments^27,36^. Ultimately, this may lead to new insights on the binding conformations and binding sites of the conformers.

## Experimental

### NMR experiments

The NMR samples were prepared by dissolving MMAE and MMAF in deuterated methanol to give a final concentration of 15.5 mg/ml. The NMR experiments were carried out at 22 °C on an 850 MHz Bruker Avance III HD NMR spectrometer equipped with a TCI (H-C/N-D) cryogenic probe. Additional NMR experiments were recorded at 37 °C in MeOD and 22 °C with a 600 MHz Bruker Avance spectrometer equipped with a QCI (H-P/C/N-D) cryogenic probe. Standard Bruker pulse sequence programs with gradient selection were used. In the 2D TOCSY and the 2D HSQC-TOCSY experiments, dipsi2 spinlocks with durations of 180 ms and 120 ms, respectively, were used. The ^13^C multiplicity edited HSQC (edHSQC) and the 2D HSQC-TOCSY spectra were acquired using echo/antiecho-TPPI gradient selection, ^1^ *J*_CH_ of 145 Hz and adiabatic decoupling. In the HSQC, also the refocusing pulses were adiabatic. The HMBC experiments were optimised for 8 Hz (62.5 ms) long-range coupling. The mixing time in the ROESY experiments was 800 ms. All 2D spectra were zero-filled once in F1 and a π/2-shifted squared sine-bell weighting function was applied in both dimensions prior to the Fourier transformation.

The NMR spectra were processed with Bruker Topspin 3.0 and referenced to the residual solvent peaks of deuterated methanol or other internal standard.

### Computational methods

The geometries were optimised at density functional theory (DFT) level, using the hybrid Tao-Perdew-Staroverov-Scuseria functional corrected for dispersion interactions, TPSSh-D3(BJ)^41–44^, with the def2-TZVPP (for isomer energy differences) and def2-SVP (for transition states and vibrational frequencies) basis sets^45^. The transition states were located with the Woelfling reaction path method^46^ followed by trust-region image minimisation^47^. Solvation effects were accounted for with the COSMO model^48^, using a dielectric constant ε = 32.6, simulating a methanol solvent. Final electronic energies were computed at the SCS-MP2 level of theory^26,49,50^, with the def2-QZVPPD quadruple-zeta basis set augmented with diffuse functions^45,51^. Enthalpies and free energies were estimated from the harmonic vibrational frequencies, with possible low-frequency modes below 20 cm^−1^ set to 20 cm^−1^; for the relative energies between isomers, gas-phase structures and frequencies were used, while for the transition state energy analysis, solvation was used.

The accuracy of the described procedure should be rather good. The largest sources of error would be the description of the solvation energy, which is based on a continuum solvation model, and the entropic contribution to the free energies, which is based on gas-phase harmonic vibrational frequencies. We estimate the relative energies between conformers to be wrong by no more than 10 kJ/mol.

An additional conformer search was performed for both MMAE and MMAF by running 200 ps classical molecular dynamics runs at 1000 K after a 10 ps heating time, using the MM+ force field based on MM2^52^. Snapshot structures were optimised quantum mechanically at 1 ps intervals. All quantum chemical calculations were performed with the Turbomole program package^53–57^, the molecular dynamics were performed with Hyperchem.

## Electronic supplementary material

Supplementary information
