# Supplementary material for: New insight on the structural features of the cytotoxic auristatins MMAE and MMAF revealed by combined NMR spectroscopy and quantum chemical modelling
Source: Sci Rep. 2017 Nov 21;7:15920. doi: 10.1038/s41598-017-15674-1 (PMC5698355; doi:10.1038/s41598-017-15674-1)
Supplement: Supplementary file 1 — Supplementary information [file 41598_2017_15674_MOESM1_ESM.pdf]

Supplementary information

New insight on the structural features of the common cytotoxic auristatins MMAE and MMAF revealed by combined NMR spectroscopy and quantum chemical modelling

Mikael P. Johansson,[a] Hannu Maaheimo[b] & Filip S. Ekholm*[a,c]

[a] Department of Chemistry, University of Helsinki, PO Box 55, A. I. Virtasen aukio 1, 00014 Helsinki, Finland. Email: filip.ekholm@helsinki.fi

[b] VTT Technical Research Centre of Finland Ltd, PO Box 1000, 02044 VTT, Finland

[c] Glykos Finland Ltd, Viikinkaari 6, 00790 Helsinki, Finland

NMR spectroscopic characterisation of MMAE

The sole aromatic residues in **1A** (*cis*-conformer) and **1B** (*trans*-conformer) were selected as suitable starting points for the NMR spectroscopic characterisation of MMAE. In the carbon spectrum, the C-1 (1) and C-1′ (1′) signals are well-resolved and appear at 144.1 (**1A**) and 143.9 (**1B**) ppm. In the HMBC spectrum (Figure 5), the cross peaks from C-1 (1) to H-3 (1) and H-5 (1) at 7.34 ppm, H-7 (1) at 4.52 ppm and H-8 (1) at 4.24 ppm were visible. There were no HMBC correlations between C-1 (1) and H-2 (1) or H-6 (1). The conventional use of edHSQC (Figure 3) and COSY (not shown) resulted in the identification of all signals in residue (1). The signals of residue (1′) were identified and assigned in a similar fashion. It should be noted that all of the ^{1}H- and ^{13}C chemical shifts in residues (1) and (1′) are similar. In fact, they differ by less than 0.1 ppm in the ^{1}H-NMR spectrum and 1.0 ppm in the ^{13}C-NMR spectrum.

The norephedrine-dolaproine amide bonds (C-1 (2) at 175.5 ppm and C-1′ (2′) at 175.7 ppm) and the protons close to them (H-8 (1) at 4.24 ppm, H-2 (2) at 2.12 ppm, 2-CH_3 (2) at 1.18 ppm and H-8′ (1′) at 4.20 ppm, H-2′ (2′) at 2.22 ppm, 2′-CH_3 (2′) at 1.12 ppm) were identified based on the cross peaks in the HMBC spectrum. The other signals in dolaproine residues (2) and (2′) could be identified by an iteration of this approach. The edHSQC-method was found to be a good tool for further verification of the assignments since CH/CH_3-protons and CH_2-protons appear in separate phases. In the dolaproine residues, many of the chemical shifts were similar, however, positions 3, 3′, 4, 4′, 7 and 7′ were found to differ significantly. The chemical shift difference was found to be greatest for position 3 (H-3 (2) at 3.42 ppm, C-3 (2) at 86.6 ppm) and 3′ (H-3′ (2′) at 3.87 ppm, C-3′ (2′) at 83.5 ppm). The deviation for H-4 (2) and H-4′ (2′) was identical *id.est.* 0.44 ppm, however, the corresponding carbon signals C-4 (2) and C-4′ (2′) appeared at a similar frequency. The H-7′a (2′) and H-7′b (2′) signals were separated by 0.17 ppm while the H-7a (2) and H-7b (2) were separated by 0.49 ppm.

With the chemical shifts of residues (2) and (2′) obtained, the next step was to identify the dolaproine-dolaisoleuine amide bonds (C-1 (3), C-1′ (3′)) and the other signals on residues (3) and (3′). The HMBC correlations of H-4 (2) and H-4′ (2′) were weak and the H-4′ (2′)/C-1′ (3′) cross peak could barely be observed while the H-4 (2)/C-1 (3) correlation was absent. The HMBC correlations between H-7a (2) (3.68 ppm)/H-7b (2) (3.19 ppm)/C-1 (3) (171.8 ppm) were utilized to acquire a starting point for residue (3). A similar protocol was utilized to confirm the chemical shift of C-1′ (3′) at 171.7 ppm. From this point forward, all of the ^{1}H- and ^{13}C-chemical shifts in residues (3) and (3′) could be identified and assigned by analyzing the spectra acquired with the various NMR spectroscopic techniques.

Continuing on the NMR spectroscopic characterisation, we examined the HMBC correlations of H-4 (3) (4.88 ppm) and H-4′ (3′) (4.76 ppm). The corresponding HMBC correlations to C-1 (4) (174.5 ppm) and C-1′ (4′) (174.7 ppm) were identified. In addition, the HMBC cross peaks between C-1 (4)/(3)/(4)-N-CH_3 and C-4 (3)/(3)/(4)-N-CH_3 were visible and could be utilized to confirm the assignment up to this point (in both rotamers). Assigning the remaining signals in residues (4) and (4′) was accomplished by the use of the standard protocol featuring HMBC, edHSQC, COSY, TOCSY, HSQC-TOCSY.

As expected the remaining signals in the ^{1}H- and ^{13}C-NMR spectra could be assigned to residues (5) and (5′). For example, H-2 (5) at 3.70 ppm and H-2 (4) at 4.80 ppm had HMBC correlations to C-1 (5) at 167.4 ppm thus assuring that all residues had been assigned correctly (the same patterns were identified in (5′)). The chemical shifts for the H-2 (5) (3.70 ppm) and H-2′ (5′) (3.68 ppm) protons were significantly different than those reported for the corresponding protons in dolastatin 10 (2.65 ppm and 2.39 ppm).

While not mentioned previously, the commercial MMAE utilized in this study was supplied as a TFA-salt. The signals from TFA are not listed in the tables but appeared in the carbon spectrum as a quartet at 162.3 ppm ($J_{C,F}$ = 35.8 Hz) and a quartet at 117.9 ppm ($J_{C,F}$ = 291.8 Hz). In addition to these peaks, there was an unidentified signal at 101.4 ppm without edHSQC and HMBC correlations.

NMR spectroscopic characterisation of MMAF

The NMR spectroscopic characterisation of MMAF will not be discussed in detail since the guidelines provided above are directly applicable to MMAF. Instead, the chemical shift values of **2A** (*cis*-conformer) and **2B** (*trans*-conformer) will be compared to each other and those observed for MMAE in order to uncover common trends for this class of compounds. For this comparison to be possible, the spectra of MMAF were also measured in deuterated methanol. It should be noted that MMAF was supplied as a TFA-salt. The chemical shifts of TFA are not listed in tables 3 and 4. In the ^{13}C-spectrum of MMAF, there is two different sets of signals for TFA which might reflect the existence of the two well-known isomers, two deviating salt forms or alternatively free TFA in addition to the salt. These signals appear at 167.7 ppm (q, $J_{C,F}$ = 35 Hz), 158.9 ppm (q, $J_{C,F}$ = 42 Hz), 118.0 ppm (q, $J_{C,F}$ = 292.4 Hz) and 116.0 ppm (q, $J_{C,F}$ = 284.4 Hz). In the spectra of MMAF, there is also an unknown signal at 101.4 ppm without HMBC or HSQC cross peaks.

The C-1 (1) (138.7 ppm) and C-1′ (1′) (138.7 ppm) were the starting points utilized in the NMR spectroscopic characterisation of MMAF (HMBC and edHSQC spectra showed in Figure 12 and 13). In the phenylalanine residue, the chemical shifts were fairly similar for both **2A** and **2B**. A direct comparison to the chemical shifts of MMAE is not warranted since this residue is different. In the dolaproine residues (2) and (2′), the chemical shifts followed a similar pattern as reported for MMAE above *id. est.* C-3 (2) (86.8 ppm) appeared 3.5 ppm downfield from C-3′ (2′) (83.3 ppm) and H-3 (2) (3.41 ppm) 0.44 ppm upfield from H-3′ (2′) (3.85 ppm). A similar pattern was observed for H-4 (2)/H-4′ (2′) although the effect was reduced ($\Delta\delta$ = 0.31 ppm) when compared to the values of H-3 (2)/H-3′ (2′). The previously described pattern concerning the chemical shift difference between H-7a (2) and H-7b (2)/H-7′a (2′) and H-7′b (2′) was also observed in the spectra of MMAF ($\Delta\delta$ = 0.46 ppm in (2), 0.17 ppm in (2′)). Since these signals show a similar pattern in both auristatins and the previously reported data on dolastatin 10, it can be concluded that these are general NMR spectroscopic trends for this class of compounds. Apart from these signals, the remaining chemical shifts in (2) and (2′) were similar. In residues (3) and (3′), the H-2 (3) protons were split into one d and one dd, while the H-2′ (3′) protons appeared together as a d (as also observed for MMAE). Apart from these minor deviations, all of the remaining signals in **2A** and **2B** appeared at similar chemical shifts. It should be noted that the H-2 (5) and H-2′ (5′) protons appear at a similar chemical shift (~3.70 ppm) in deuterated methanol in both MMAE and MMAF. This shift is considerably different than the values

reported for dolastatin 10 in CD_2Cl_2 (2.39 ppm), DMSO-d6 (2.65 ppm) and CD_3OD (3.05 ppm) thus reflecting a deviation in the structural features of auristatins when compared to dolastatin 10.

Supplementary Table 1. Summary of the NMR results of **1A** (*cis*-conformer) measured at 22 °C in CD_3OD with a Bruker 850 MHz instrument. Chemical shifts are expressed in ppm using solvent residual peaks as an internal reference (3.31 ppm δ ^{1}H and 49.0 ppm δ ^{13}C). Coupling patterns are given as d (doublet), t (triplet), q (quartet), m (multiplet) etc. and coupling constants are provided only once when first encountered.

POSITION	δ ^{13}C	δ ^{1}H (*J*, HZ)	HMBC (H → C)-CORRELATIONS	ROESY CORRELATIONS[A]
**1 (1)**	**144.1**	-	-	-
2 (1)	128.1	7.39 (dd, $J_{2,4}$ = 1.6, $J_{2,3}$ = 7.8 Hz)	C-3 (1), C-5 (1)	H-7 (1), H-8 (1)
3 (1)	129.8	7.34 (ap t, $J_{3,4}$ = 7.3 Hz)	C-1 (1), C-2 (1), C-4 (1), C-6 (1)	not determined
4 (1)	128.6	7.22 (ap tt)	C-2 (1), C-6 (1)	not determined
5 (1)	129.8	7.34 (ap t, identical with H-3 (1))	C-1 (1), C-2 (1), C-4 (1), C- 6 (1)	not determined
6 (1)	128.1	7.39 (ap dd, identical with H-2 (1))	C-3 (1), C-5 (1)	H-7 (1), H-8 (1)
7 (1)	77.5	4.52 (d, $J_{7,8}$ =7.7 Hz)	C-1 (1), C-2 (1), C-6 (1), C-8 (1), 8-CH_3 (1)	H-2 (1), H-6 (1), H-8 (1), 8-CH_3 (1)
8 (1)	50.7	4.24 (dq, $J_{8,8\text{-}CH3}$ = 6.5 Hz)	C-1 (1), C-7 (1), 8-CH_3 (1), C-1 (2)	H-2 (1), H-6 (1), H-7 (1), 8-CH_3 (1)
8-CH_3 (1)	17.0	1.20 (d)	C-7 (1), C-8 (1)	H-7 (1), H-8 (1)
**1 (2)**	**175.5**	-	-	-
2 (2)	45.4	2.12 (dq, $J_{2,2\text{-}CH3}$ = 6.8 Hz, $J_{2,3}$ = 10.0 Hz)	C-1 (2), C-3 (2), 2-CH_3 (2)	H-5a (2), 2-CH_3 (2)
2-CH_3 (2)	15.8	1.18 (d)	C-1 (2), C-2 (2), C-3 (2)	H-2 (2), H-3 (2)
3 (2)	86.6	3.42 (dd, $J_{3,4}$ = 1.2 Hz)	C-2 (2), 2-CH_3 (2), 3-OCH_3 (2), C-4 (2), C-5 (2)	H-2 (2), 2-CH_3 (2), H-2a (3)
3-OCH_3 (2)	62.0	3.34 (s)	C-3 (2)	2-CH_3 (2)
4 (2)	60.6	3.27 (m)	C-3 (2) weak, C-5 (2) weak, C-6 (2) weak, C-7 (2) weak	H-2b (3), H-5b (2)
5 (2)	26.6	1.78 (5a) (m)<br>1.37 (5b) (m)	C-3 (2), C-4 (2), C-6 (2), C-7 (2)	H-5a: H-2 (2), H-5b (2)<br>H-5b: H-4 (2), H-5a (2)
6 (2)	24.4	1.88 (6a) (m)<br>1.57 (6b)	C-4 (2), C-5 (2), C-7 (2)	H-6a: H-6b (2), H-7b (2)<br>H-6b: H-6a (2)

		(m)		
7 (2)	48.1	3.68 (7a) (m) 3.19 (7b) (m)	C-4 (2), C-5 (2), C-6 (2), C-1 (3)	H-7a: H-7b (2) H-7b: H-6a (2), H-7a (2)
**1 (3)**	**171.8**	-	-	-
2 (3)	36.7	2.53 (2a) (d, $J_{2a,2b}$ = –15.8 Hz) 2.46 (2b) (dd, $J_{2b,3}$ = 10.4 Hz)	C-1 (3), C-3 (3), C-4 (3)	H-2a: H-3 (2), H-3 (3), H-5 (3), aliphatic region H-2b: not determined
3 (3)	78.8	4.19 (m)	C-1 (3), C-2 (3), 3-OCH_3 (3)	H-2a (3), 3-OCH_3 (3), aliphatic region
3-OCH_3 (3)	58.6	3.36 (s)	C-3 (3)	H-3 (3), H-4 (3)
4 (3)	57.8	4.88 (m)	C-2 (3), C-3 (3), C-5 (3), C-6 (3), 5-CH_3 (3), C-1 (4)	not observed
5 (3)	33.6	1.90 (m)	C-4 (3), 5-CH_3 (3), C-7 (3)	H-2a (3), (3)/(4)-N-CH_3, aliphatic region
5-CH_3 (3)	15.9	0.98 (d, $J_{5\text{-}CH3,5}$ = 6.5 Hz)	C-4 (3), C-5 (3), C-6 (3),	H-3 (3), H-4 (3), H-5 (3)
6 (3)	27.1	1.38 (6a) (m) 1.02 (6b) (m)	C-4 (3), C-5 (3), 5-CH_3 (3), C-7 (3)	not determined
7 (3)	10.9	0.88 (t, $J_{7,6}$ = 7.5 Hz)	C-5 (3), C-6 (3)	H-5 (3) weak, H-6a (3)
**(3)/(4)-N-CH_3**	**33.2**	**3.29 (s)**	C-4 (3), C-1 (4)	H-2 (1) and/or H-6 (1), H-2a and/or H-2b (3), H-5 (3), H-2 (4)
**1 (4)**	**174.5**	-	-	-
2 (4)	56.7	4.80 (d, $J_{2,3}$ =8.4 Hz)	C-1 (4), C-3 (4), 3-CH_3 (4), C-4 (4), C-1 (5)	(3)/(4)-N-CH_3, H-3 (4), aliphatic region
3 (4)	31.5	2.15 (dqq, $J_{3,3\text{-}CH3}$ = 6.7, $J_{3,4}$ = 6.8 Hz	C-1 (4), C-2 (4), 3-CH_3 (4), C-4 (4)	not determined
3-CH_3 (4)	18.9	0.99 (d)	C-2 (4), C-3 (4), C-4 (4)	H-2 (4), H-3 (4), aliphatic region
4 (4)	19.6	1.05 (d)	C-2 (4), C-3 (4), 3-CH_3 (4)	not determined
**1 (5)**	**167.4**	-	-	-
2 (5)	68.1	3.70 (d, $J_{2,3}$ = 5.4 Hz)	C-1 (5), C-3 (5), 3-CH_3 (5), C-4 (5), (5)-N-CH_3	H-3 (5), H-4 (5), (5)-N-CH_3
3 (5)	31.5	2.20 (dqq, $J_{3,3\text{-}CH3}$ = 6.5, $J_{3.4}$ = 6.9 Hz	C-1 (5), C-2 (5), 3-CH_3 (5), C-4 (5)	H-2 (5), aliphatic region
3-CH_3 (5)	18.3	1.04 (d)	C-2 (5), C-3 (5), C- 4 (5)	not determined

4 (5)	18.7	1.08 (d)	C-2 (5), C-3 (5), 3-CH_3 (5)	H-2 (5), H-3 (5), aliphatic region
**(5)-N-CH_3**	**33.1**	**2.68 (s)**	C-2 (5)	H-2 (5)

[A] The ROEs were not determined in crowded areas of the spectrum where the uncertainty was high, if these ROE-correlations were in the aliphatic region it is mentioned in the table.

Supplementary Table 2. Summary of the NMR results of compound **1B** (*trans*-conformer) measured at 22 °C in CD_3OD with a Bruker 850 MHz instrument. Chemical shifts are expressed in ppm using solvent residual peaks as an internal reference (3.31 ppm δ ^1H and 49.0 ppm δ ^{13}C). Coupling patterns are given as d (doublet), t (triplet), q (quartet), m (multiplet) etc. and coupling constants are provided only once when first encountered.

POSITION	δ ^{13}C	δ ^1H (J, HZ)	HMBC (H → C)-CORRELATIONS	ROESY CORRELATIONS[A]
**1′ (1′)**	**143.9**	-	-	-
2′ (1′)	127.9	7.39 (dd, $J_{2',4'}$ = 1.6, $J_{2',3'}$ = 7.8 Hz)	C-3′ (1′), C-5′ (1′)	H-7′ (1′), H-8′ (1′)
3′ (1′)	129.5	7.29 (ap t, $J_{3',4'}$ = 7.3 Hz)	C-1′ (1′), C-2′ (1′), C-4′ (1′), C-6′ (1′)	not determined
4′ (1′)	128.4	7.21 (ap tt)	C-2′ (1′), C-6′ (1′)	not determined
5′ (1′)	129.5	7.29 (ap t, identical with H-3′ (1′))	C-1′ (1′), C-2′ (1′), C-4′ (1′), C-6′ (1′)	not determined
6′ (1′)	127.9	7.39 (ap dd, identical with H-2′ (1′))	C-3′ (1′), C-5′ (1′)	H-7′ (1′), H-8′ (1′)
7′ (1′)	77.3	4.61 (d, $J_{7',8'}$ = 6.5 Hz)	C-1′ (1′), C-2′ (1′), C-6′ (1′), C-8′ (1′), 8′-CH_3 (1′)	H-2′ (1′), H-6′ (1′), 8′-CH_3 (1′)
8′ (1′)	51.4	4.20 (dq, $J_{8',8'-CH3}$ = 6.7 Hz)	C-1′ (1′), C-7′ (1′), 8′-CH_3 (1′), C-1′ (2′)	H-2′ (1′), H-6′ (1′), 8′-CH_3 (1′)
8′-CH_3 (1′)	16.0	1.15 (d)	C-7′ (1′), C-8′ (1′)	H-7′ (1′), H-8 (1′)
**1′ (2′)**	**175.7**	-	-	-
2′ (2′)	45.9	2.22 (dq, $J_{2',2'-CH3}$ = 6.8, $J_{2',3'}$ = 9.2 Hz)	C-1′ (2′), C-3′ (2′), C-4′ (2′), 2′-CH_3 (2′)	H-3′ (2′), H-4′ (2′)
2′-CH_3 (2′)	15.1	1.12 (d)	C-1′ (2′), C-2′ (2′), C-3′ (2′),	H-2′ (2′), H-3′ (2′), 3′-OCH_3 (2′)
3′ (2′)	83.5	3.87 (dd, $J_{3',4'}$ = 2.1 Hz)	C-1′ (2′), C-2′ (2′), 2′-CH_3 (2′), C-4′ (2′), 3′-OCH_3 (2′),	3′-OCH_3 (2′), H-2′ (2′), 2′-CH_3 (2′)
3′-OCH_3 (2′)	61.5	3.35 (s)	C-3′ (2′)	not determined

4′ (2′)	60.8	3.71 (m)	C-3′ (2′) weak, C-5′ (2′) weak, C-6′ (2′) weak, C-7′ (2′) weak, C-1′ (3′) weak	H-5′b (2′)
5′ (2′)	25.6	1.84 (5′a) (m) 1.58 (5′b) (m)	C-3′ (2′), C-4′ (2′), C-6′ (2′), C-7′ (2′)	H-5′a: not determined H-5′b: H-4′ (2′) H-5′a (2′)
6′ (2′)	25.9	1.95 (6′a) (m) 1.69 (6′b) (m)	C-4′ (2′), C-5′ (2′), C-7′ (2′)	H-6′a: H-6′b (2′), H-7′b (2′) H-6′b: H-6′a (2′), H-7′a (2′)
7′ (2′)	48.4	3.56(7′a) (m) 3.39 (7′b) (m)	C-4′ (2′), C-5′ (2′), C-6′ (2′), C-1′ (3′)	H-7′a: H-6′b (2′), H-2′ (3′) H-7′b: not determined
**1′ (3′)**	**171.7**	-	-	-
2′ (3′)	38.2	2.47 (d, $J_{2',3'}$ = 6.2 Hz)	C-1′ (3′), C-3′ (3′)	H-7′a (2′), H-3′ (3′), H-5′ (3′), (3′)/(4′)-N-CH_3, aliphatic region
3′ (3′)	79.7	4.08 (m)	C-1′ (3′), C-2′ (3′), 3′-OCH_3 (3′)	H-2′ (3′), 3′-OCH_3 (3′), aliphatic region
3′-OCH_3 (3′)	58.3	3.30 (s)	C-3′ (3′)	H-3′ (3′)
4′ (3′)	58.4	4.76 (m)	not determined	not determined
5′ (3′)	33.7	1.79 (m)	C-4′ (3′) weak, 5′-CH_3 (3′), C-7′ (3′)	H-2′ (3′), (3′)/(4′)-N-CH_3
5′-CH_3 (3′)	16.3	1.01 (d, $J_{5'\text{-}CH3,5'}$ = 6.8 Hz)	C-4′ (3′), C-5′ (3′), C-6′ (3′)	not determined
6′ (3′)	27.1	1.42 (6′a) (m) 1.02 (6′b) (m)	C-4′ (3′), 5′-CH_3 (3′), C-7′ (3′)	not determined
7′ (3′)	10.9	0.86 (t, $J_{7',6'}$ = 7.5 Hz)	C-5′ (3′), C-6′ (3′)	H-5′ (3′), H-6′a (3′)
**(3′)/(4′)-N-CH_3**	**33.0**	**3.14 (s)**	C-4′ (3′), C-1′ (4′)	H-2′ (3′), H-3′ (3′), H-5′ (3′), H-6′a (3′), H-2′ (4′), aliphatic region
**1′ (4′)**	**174.7**	-	-	-
2′ (4′)	56.9	4.70 (d, $J_{2',3'}$ =8.8 Hz)	C-1′ (4′), C-3′ (4′), 3′-CH_3 (4′), C-4′ (4′), C-1′ (5′)	(3′)/(4′)-N-CH_3, H-3′ (4′), aliphatic region
3′ (4′)	31.9	2.08 (dqq, $J_{3',3'\text{-}CH3}$ = 6.7, $J_{3',4'}$ = 6.8 Hz)	C-2′ (4′), 3′-CH_3 (4′), C-4′ (4′)	not determined
3′-CH_3 (4′)	19.2	1.05 (d)	C-3′ (4′), C-4′ (4′)	not determined

4′ (4′)	19.2	1.03 (d)	C-2′ (4′), C-3′ (4′), 3′-CH_3 (4′)	not determined
**1′ (5′)**	**167.5**	-	-	-
2′ (5′)	68.0	3.68 (d, $J_{2′,3′}$ = 5.5 Hz)	C-1′ (5′), C-3′ (5′), 3′-CH_3 (5′), C-4′ (5′), (5′)-N-CH_3	H-3′ (5′), (5′)-N-CH_3, aliphatic region
3′ (5′)	31.5	2.18 (dqq, $J_{3′,3′\text{-}CH3}$ = 6.7 Hz, $J_{3′,4′}$ = 6.8 Hz)	C-2′ (5′), 3′-CH_3 (5′), C-4′ (5′)	H-2′ (5′), aliphatic region
3′-CH_3 (5′)	18.7	1.02 (d)	C-2′ (5′), C-3′ (5′), C-4′ (5′)	not determined
4′ (5′)	18.3	1.05 (d)	C-2′ (5′), C-3′ (5′), 3′-CH_3 (5′)	not determined
**(5′)-N-CH_3**	**33.1**	**2.65 (s)**	C-2′ (5′)	H-2′ (5′), aliphatic region

[A] The ROEs were not determined in crowded areas of the spectrum where the uncertainty was high, if these ROE-correlations were in the aliphatic region it is mentioned in the table.

Supplementary Table 3. Summary of the NMR results of compound **2A** (*cis*-conformer) measured at 22 °C in CD_3OD with a Bruker 850 MHz instrument. Chemical shifts are expressed in ppm using solvent residual peaks as an internal reference (3.31 ppm δ ^{1}H and 49.0 ppm δ ^{13}C). Coupling patterns are given as d (doublet), t (triplet), q (quartet), m (multiplet) etc. and coupling constants are provided only once when first encountered.

POSITION	δ ^{13}C	δ ^{1}H (*J*, HZ)	HMBC (H → C)-CORRELATIONS	ROESY CORRELATIONS[A]
**1 (1)**	**138.7**	-	-	-
2 (1)	130.0	7.29–7.23 (m)	C-3 (1), C-4 (1), C-5 (1), C-7 (1)	not determined
3 (1)	129.6	7.29–7.23 (m)	not determined	not determined
4 (1)	127.8	7.19 (tt, $J_{4,2}$ = 1.4, $J_{4,3}$ = 7.4 Hz	C-2 (1), C-3 (1), C-5 (1), C-6 (1)	not determined
5 (1)	129.6	7.29–7.23 (m, identical to H-3(1))	not determined	not determined
6 (1)	130.0	7.29–7.23 (m, identical to H-2 (1))	C-3 (1), C-4 (1), C-5 (1), C-7 (1)	not determined
7 (1)	37.9	3.33 (H-7a) (dd) 2.92 (H-7b) (dd)	C-1 (1), C-2 (1), C-6 (1), C-8 (1), 8-CO_2H (1)	H-7a: H-7b (1), aromatic region H-7b: H-7a (1), aromatic region
8 (1)	53.7	4.79 (dd, *J* = 4.3, 11.7 Hz)	C-1 (1), C-7 (1), 8-CO_2H (1), C-1 (2)	not determined
8-CO_2H (1)	174.8	-	-	-
**1 (2)**	**176.6**	-	-	-
2 (2)	45.3	2.25 (dq, $J_{2,2\text{-}CH3}$ = 6.8, $J_{2,3}$ = 9.7 Hz)	C-1 (2), 2-CH_3 (2), C-3 (2), C-4 (2)	H-3 (2), H-5a (2)
2-CH_3 (2)	15.6	1.20	C-2 (2), C-3 (2)	H-2 (2), H-3 (2)

		(d)		
3 (2)	86.8	3.41 (br d)	C-2 (2), 2-CH_3 (2), C-(4), C-5 (2)	H-2 (2), 2-CH_3 (2), H-2b (3)
3-OCH_3 (2)	62.1	3.35 (s)	C-3 (2)	not determined
4 (2)	60.6	3.35 (m)	C-5 (2)	not determined
5 (2)	26.5	1.77 (H-5a) (m) 1.27 (H-5b) (m)	C-3 (2), C-4 (2), C-6 (2), C-7 (2)	H-5a: H-2 (2), H-4 (2), H-5b (2) H-5b: not determined
6 (2)	24.5	1.87 (H-6a) (m) 1.54 (H-6b) (m)	C-4 (2), C-5 (2), C-7 (2)	not determined
7 (2)	48.0	3.66 (H-7a) (m) 3.20 (H-7b) (m)	C-5 (2), C-6 (2), C-1 (3)	H-7a: H-7b (2) H-7b: H-7a (2)
**1 (3)**	**172.1**	-	-	-
2 (3)	36.7	2.47 (H-2a) (d, $J_{2a,2b}$ = –15.2 Hz) 2.42 (H-2b) (dd, $J_{2b,3}$ = 10.5 Hz)	C-1 (3), C-3 (3)	H-2a: H-5 (3) H-2b: H-4 (2), (3)/(4)-N-CH_3
3 (3)	79.1	4.15 (m)	C-1 (3), C-2 (3), C-4 (3)	H-2 (3), H-4 (3), aliphatic region
3-OCH_3 (3)	58.5	3.34 (s)	C-3 (3)	not determined
4 (3)	57.9	4.83 (m)	C-2 (3), C-3 (3), C-5 (3), 5-CH_3 (3), C-6 (3), C-1 (4)	not determined
5 (3)	33.5	1.87 (m)	C-4 (3), 5-CH_3 (3), C-7 (3)	H-3 (3)
5-CH_3 (3)	15.9	0.98 (d, $J_{5\text{-CH3},5}$ = 6.6 Hz)	C-4 (3), C-5 (3), C-6 (3)	not determined
6 (3)	27.0	1.40 (H-6a) (m) 1.00 (H-6b) (m)	C-5 (3), 5-CH_3 (3), C-7 (3)	not determined
7 (3)	10.9	0.87 (t, $J_{7,6}$ = 7.4 Hz)	C-5 (3), C-6 (3)	not determined
**(3)/(4)-N-CH_3**	**33.1**	**3.23 (s)**	C-4 (3), C-1 (4)	H-2 (4), H-2b (3), aromatic region
**1 (4)**	**174.6**	-	-	-
2 (4)	56.7	4.82 (d, $J_{2,3}$ = 8.0 Hz)	C-1 (4), C-3 (4), C-4 (4), C-1 (5)	(3)/(4)-N-CH_3
3 (4)	31.7	2.18 (dqq, $J_{3,4}$ = 6.7, $J_{3,3\text{-CH3}}$ = 6.8 Hz)	C-1 (4), C-2 (4), C-4 (4)	not determined
3-CH_3 (4)	18.9	1.05 (d)	C-2 (4), C-3 (4), C-4 (4)	not determined

4 (4)	19.8	1.12 (d)	C-2 (4), C-3 (4), 3-CH_3 (4)	H-2 (4), (3)/(4)-N-CH_3, aromatic region
**1 (5)**	**167.6**	**-**	**-**	**-**
2 (5)	68.1	3.71 (d, $J_{2,3}$ = 5.1 Hz)	C-1 (5), 5-N-CH_3, C-3 (5), C-4 (5), 3-CH_3 (5)	H-3 (5), 5-N-CH_3, aliphatic region
3 (5)	31.5	2.20 (dqq, $J_{3,4}$ = 7.0 Hz, $J_{3,3\text{-}CH3}$ = 7.0 Hz	C-1 (5), C-2 (5), C-4 (5), 3-CH_3 (5)	aliphatic region
3-CH_3 (5)	18.3	1.04 (d)	C-2 (5), C-3 (5), C-4 (5)	not determined
4 (5)	18.8	1.07 (d)	C-2 (5), C-3 (5), 3-CH_3 (5)	not determined
**(5)-N-CH_3**	**33.1**	**2.67 (s)**	C-2 (5)	not determined

[A] The ROEs were not determined in crowded areas of the spectrum where the uncertainty was high, if these ROE-correlations were in the aliphatic region it is mentioned in the table.

Supplementary Table 4. Summary of the NMR results of compound **2B** (*trans*-conformer) measured at 22 °C in CD_3OD with a Bruker 850 MHz instrument. Chemical shifts are expressed in ppm using solvent residual peaks as an internal reference (3.31 ppm δ ^1H and 49.0 ppm δ ^{13}C). Coupling patterns are given as d (doublet), t (triplet), q (quartet), m (multiplet) etc. and coupling constants are provided only once when first encountered.

POSITION	δ ^{13}C	δ ^{1}H (*J*, HZ)	HMBC (H $\rightarrow$ C)-CORRELATIONS	ROESY CORRELATIONS[A]
**1′ (1′)**	**138.7**	**-**	**-**	**-**
2′ (1′)	130.1	7.29 – 7.23 (m)	C-3′ (1′), C-4′ (1′), C-5′ (1′), C-7′ (1′)	not determined
3′ (1′)	129.5	7.29 – 7.23 (m)	not determined	not determined
4′ (1′)	127.7	4.17 (tt, $J_{4',2'}$ = 1.6, $J_{3',4'}$ = 7.1 Hz)	C-2′ (1′), C-3′ (1′), C-5′ (1′), C-6′ (1′)	not determined
5′ (1′)	129.5	7.29 – 7.23 (m, identical to H-3′ (1′)	not determined	not determined
6′ (1′)	130.1	7.29 – 7.23 (m, identical to H-2′ (1′)	C-3′ (1′), C-4′ (1′), C-5′ (1′), C-7′ (1′)	not determined
7′ (1′)	37.9	3.28 (H-7′a) (dd) 2.93 (H-7′b) (dd)	C-1′ (1′), C-2′ (1′), C-6′ (1′), C-8′ (1′), 8′-CO_2H (1′)	not determined
8′ (1′)	54.5	4.72 (dd, *J* = 4.4, 11.0 Hz)	C-1′ (1′), C-7′ (1′), 8′-CO_2H (1′), C-1′ (2′)	not determined
8′-CO_2H (1)	174.8	-	-	-
**1′ (2′)**	**176.7**	**-**	**-**	**-**
2′ (2′)	45.3	2.31 (dq, $J_{2',2'\text{-}CH3}$ = 6.8, $J_{2',3'}$ = 8.9 Hz)	C-1′ (2′), C-3′ (2′), C-4′ (2′), 2′-CH_3 (2′)	2′-CH_3 (2′), H-3′ (2′), H-5′a (2′)

2′-CH$_3$ (2′)	14.9	1.15 (d)	C-1′ (2′), C-2′ (2′), C-3′ (2′)	H-2′ (2′), H-3′ (2′)
3′ (2′)	83.3	3.85 (dd, $J_{3′,4′}$ = 1.7 Hz)	C-2′ (2′), 2′-CH$_3$ (2′), C-4′ (2′), C-5′ (2′) 3′-OCH$_3$ (2′)	H-2′ (2′), 2′-CH$_3$ (2′)
3′-OCH$_3$ (2′)	61.4	3.30 (s)	C-3′ (2′)	not determined
4′ (2′)	60.6	3.66 (m)	C-3′ (2′), C-5′ (2′), C-1′ (3′)	not determined
5′ (2′)	25.5	1.73 (H-5′a) (m)<br>1.41 (H-5′b) (m)	C-3′ (2′), C-4′ (2′), C-6′ (2′), C-7′ (2′)	H-5′a: H-2′ (2′), H-7′b (2′)<br>H-5′b: not determined
6′ (2′)	25.8	1.89 (H-6′a) (m)<br>1.61 (H-6′b) (m)	C-4′ (2′), C-5′ (2′), C-7′ (2′)	H-6′a: not determined<br>H-6′b: H-6′a (2′), H-7′a (2′)
7′ (2′)	49.0	3.52 (H-7′a) (m)<br>3.35 (H-7′b) (m)	C-4′ (2′), C-5′ (2′), C-6′ (2′), C-1′ (3′)	H-7′a: H-6′b (2′), H-2′ (3′), (3′)/(4′)-N-CH$_3$<br>H-7′b: not determined
**1′ (3′)**	**171.6**	-	-	-
2′ (3′)	38.2	2.46 (d, $J_{2′,3′}$ = 6.1 Hz)	C-1′ (3′), C-3′ (3′), C-4′ (3′)	H-7′a (2′), H-5′ (3′), (3′)/(4′)-N-CH$_3$
3′ (3′)	79.6	4.07 (m)	C-1′ (3′), C-2′ (3′), C-4′ (3′)	H-2′ (3′), H-4′ (3′)
3′-OCH$_3$ (3′)	58.3	3.29 (s)	C-3′ (3′)	not determined
4′ (3′)	58.3	4.76 (m)	C-2′ (3′)	H-3′ (3′)
5′ (3′)	33.7	1.79 (m)	not determined	H-2′ (3′)
5′-CH$_3$ (3′)	16.2	1.01 (d, $J_{5′\text{-CH3},5′}$ = 6.5 Hz)	C-4′ (3′), C-5′ (3′), C-6′ (3′)	not determined
6′ (3′)	27.1	1.40 (H-6a) (m)<br>1.00 (H-6b) (m)	not determined	not determined
7′ (3′)	10.8	0.86 (t, $J_{7′,6′}$ = 7.9 Hz)	C-5′ (3′), C-6′ (3′)	not determined
**(3′)/(4′)-N-CH$_3$**	**33.1**	**3.13 (s)**	C-4′ (3′), C-1′ (4′)	H-2′ (3′), H-3′ (3′), H-5′ (3′), H-2′ (4′)
**1′ (4′)**	**175.0**	-	-	-
2′ (4′)	56.9	4.70 (d, $J_{2′,3′}$= 8.7 Hz)	C-1′ (4′), C-3′ (4′), C-4′ (4′), 3′-CH$_3$ (4′), C-1′ (5′)	H-3′ (4′), (3′)/(4′)-N-CH$_3$
3′ (4′)	31.8	2.08 (dqq, $J_{3′,3′\text{-CH3}}$ = 6.6, $J_{3′,4′}$ = 6.9 Hz)	C-1′ (4′), C-2′ (4′), C-4′ (4′), 3′-CH$_3$ (4′)	not determined
3′-CH$_3$ (4′)	19.2	1.03	C-2′ (4′), C-3′ (4′)	not determined

		(d)		
4′ (4′)	19.1	1.05 (d)	C-2′ (4′), C-3′ (4′)	not determined
**1′ (5′)**	**167.6**	**-**	-	-
2′ (5′)	68.0	3.68 (d, $J_{2',3'}$ = 5.2 Hz)	C-1′ (5′), C-3′ (5′), C-4′ (5′), 3′-CH_3 (5′), (5′)-N-CH_3	H-3′ (5′), (5′)-N-CH_3
3′ (5′)	31.5	2.18 (dqq, $J_{3',3'-CH3}$ = 6.8 Hz, $J_{3',4'}$ = 6.9 Hz)	C-1′ (5′), C-2′ (5′), C-4′ (5′), 3′-CH_3 (5′)	not determined
3′-CH_3 (5′)	18.3	1.01 (d)	C-2′ (5′), C-3′ (5′), C-4′ (5′)	not determined
4′ (5′)	18.7	1.05 (d)	C-2′ (5′), C-3′ (5′), 3′-CH_3 (5′)	not determined
**(5′)-N-CH_3**	**33.1**	**2.65**	C-2′ (5′)	H-2′ (5′)

[A] The ROEs were not determined in crowded areas of the spectrum where the uncertainty was high, if these ROE-correlations were in the aliphatic region it is mentioned in the table.

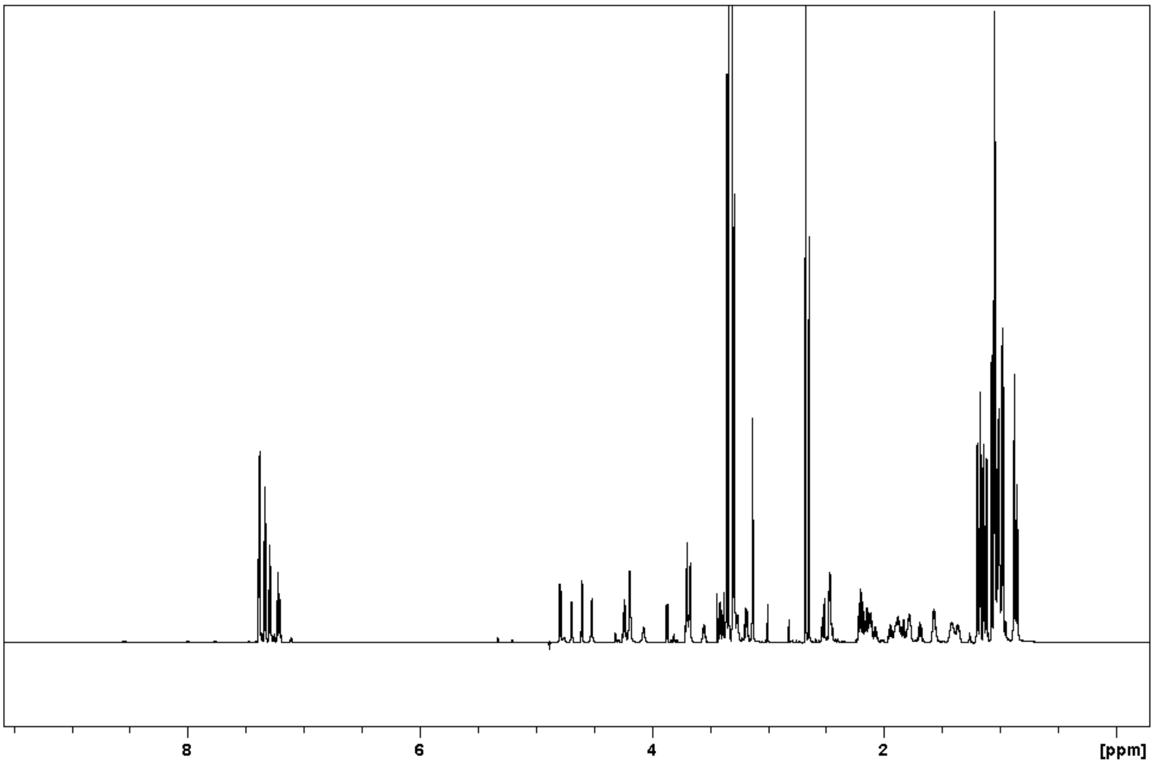

Supplementary Figure 1. ^{1}H-NMR spectrum of MMAE in CD_3OD recorded at 22 °C (850 MHz).

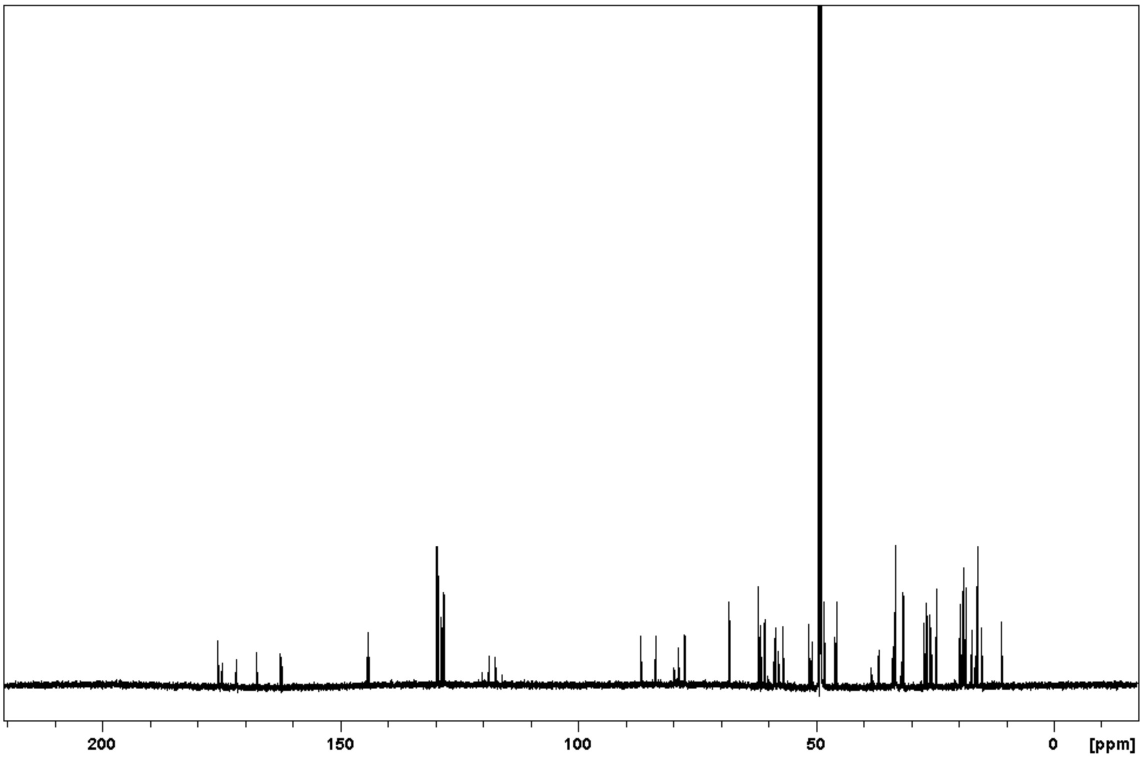

Supplmentary Figure 2. ^{13}C-NMR spectrum of MMAE in CD_3OD recorded at 22 °C (213 MHz).

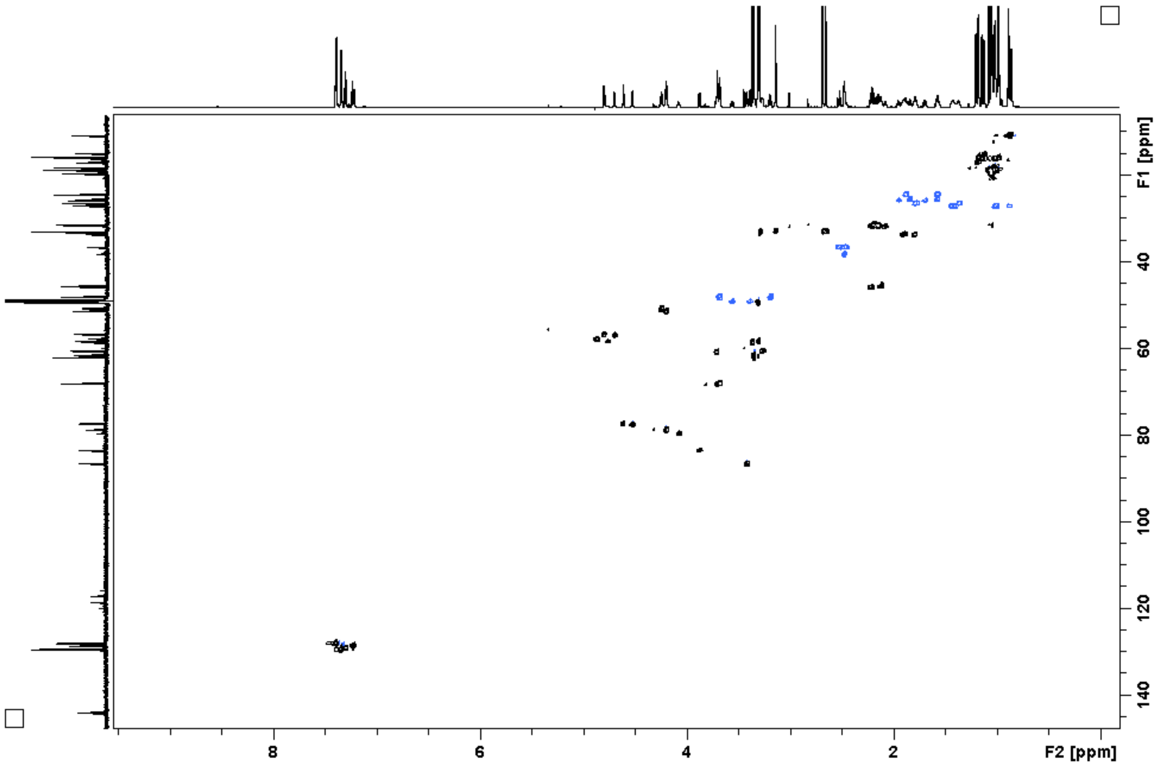

Supplementary Figure 3. EdHSQC spectrum of MMAE in CD_3OD recorded at 22 °C (850 MHz).

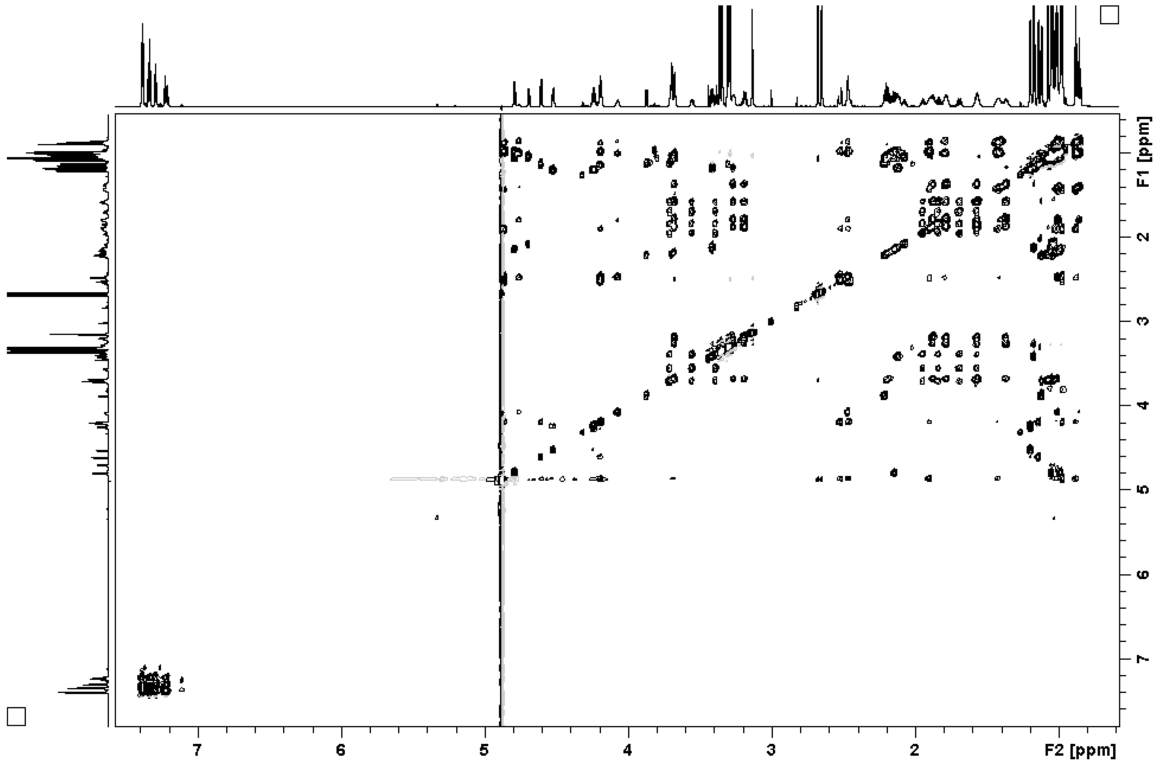

Supplementary Figure 4. 2D TOCSY spectrum of MMAE in CD_3OD recorded at 22 °C (180 ms spinlocktime) (850 MHz).

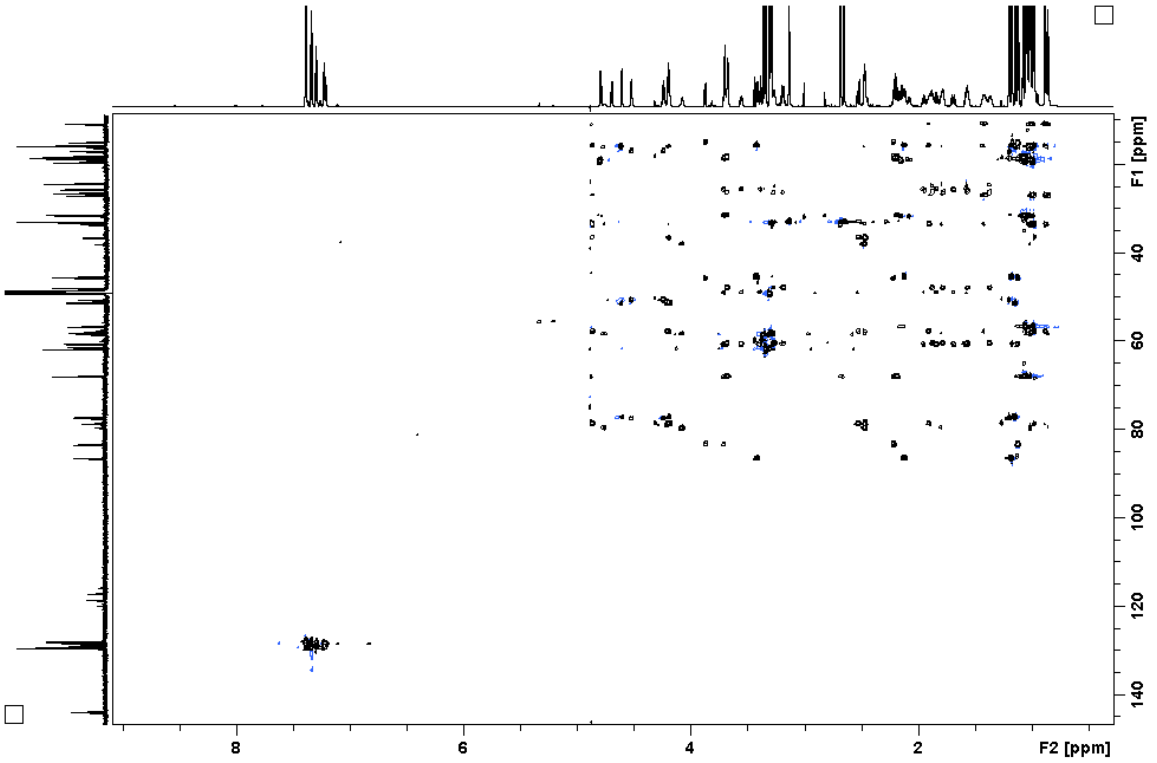

Supplementary Figure 5. 2D HSQC-TOCSY spectrum of MMAE in CD_3OD recorded at 22 °C (850 MHz).

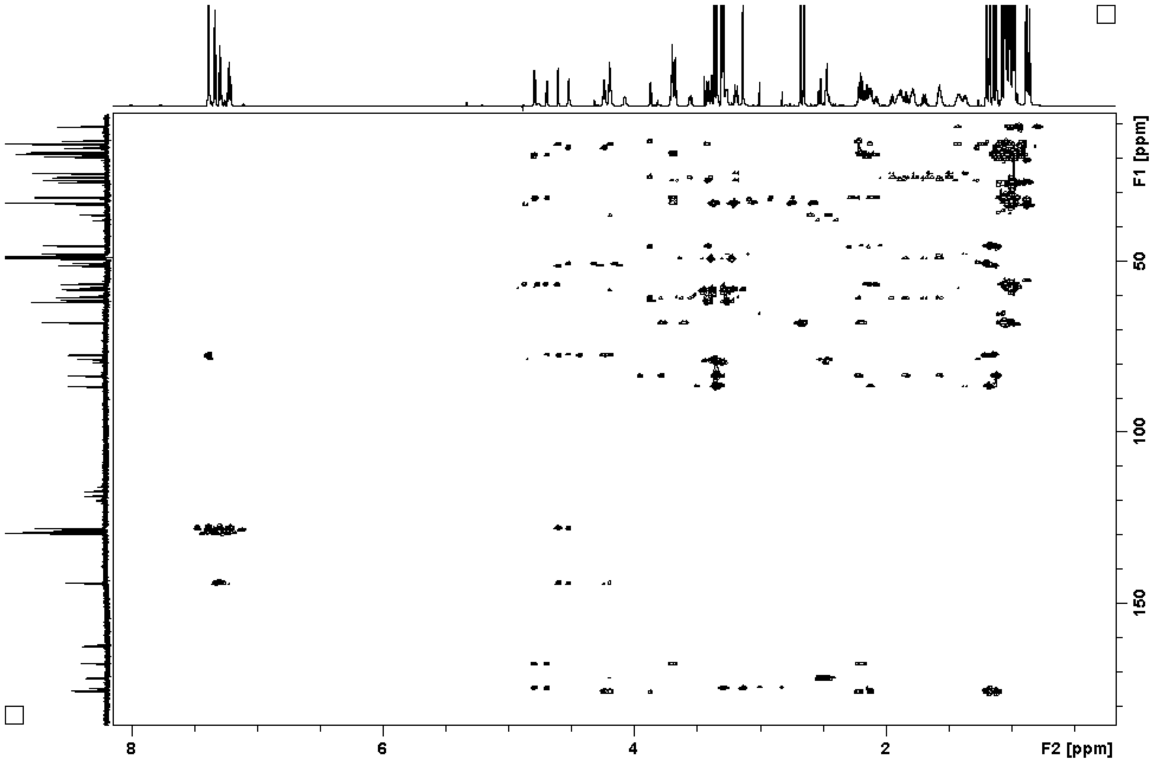

Supplementary Figure 6. HMBC spectrum of MMAE in CD_3OD recorded at 22 °C (850 MHz).

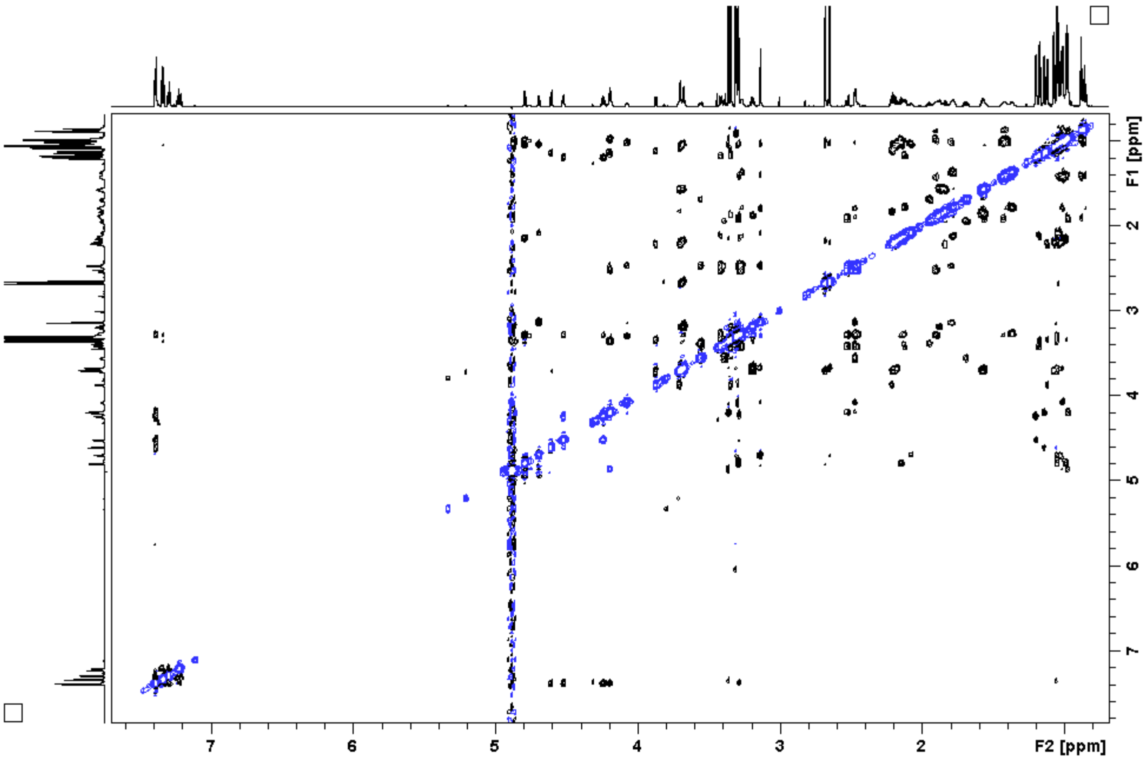

Supplementary Figure 7. 2D ROESY spectrum of MMAE in CD_3OD recorded at 22 °C (0.8 s mixing time) (850 MHz).

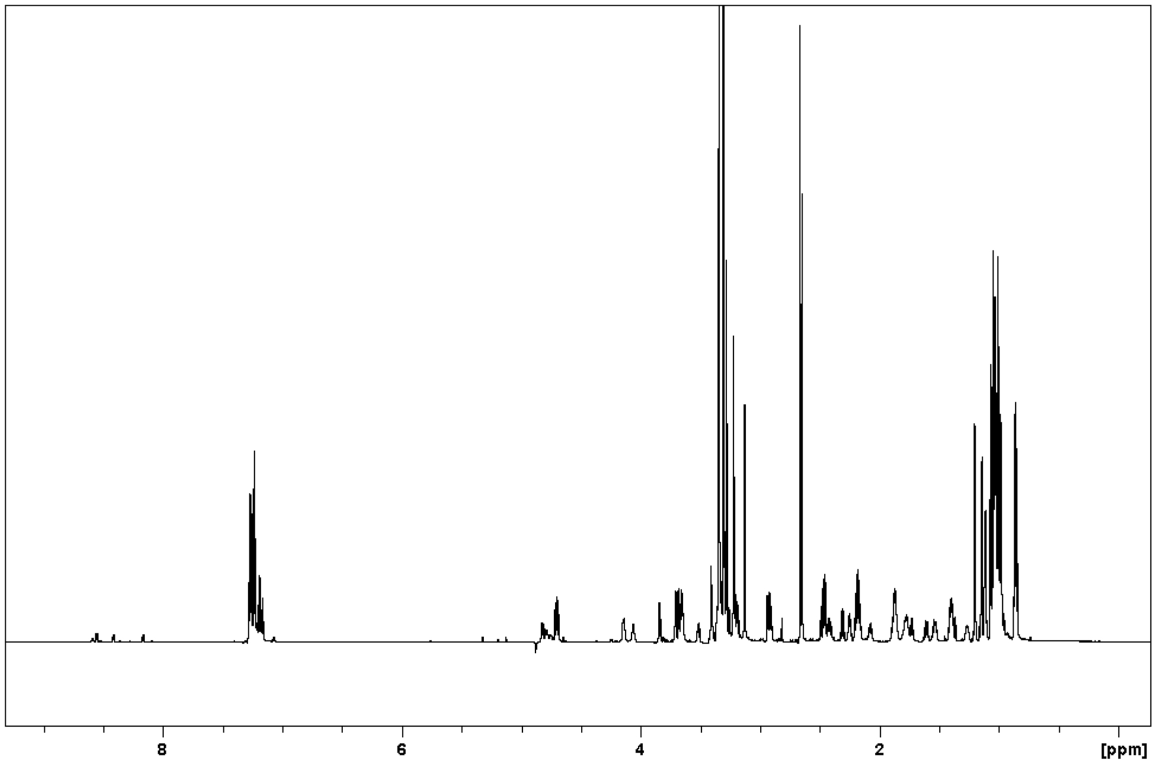

Supplementary Figure 8. ^{1}H-NMR spectrum of MMAF in CD_3OD recorded at 22 °C (850 MHz).

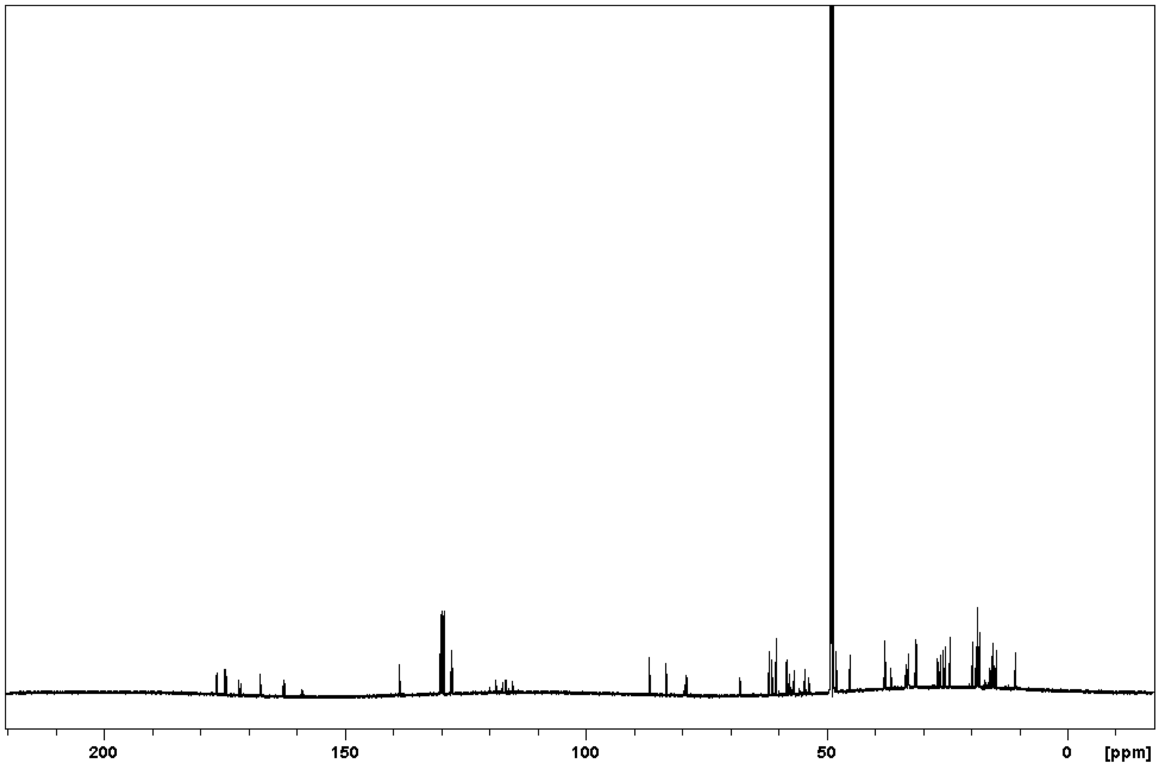

Supplementary Figure 9. ^{13}C-NMR spectrum of MMAF in CD_3OD recorded at 22 °C (213 MHz).

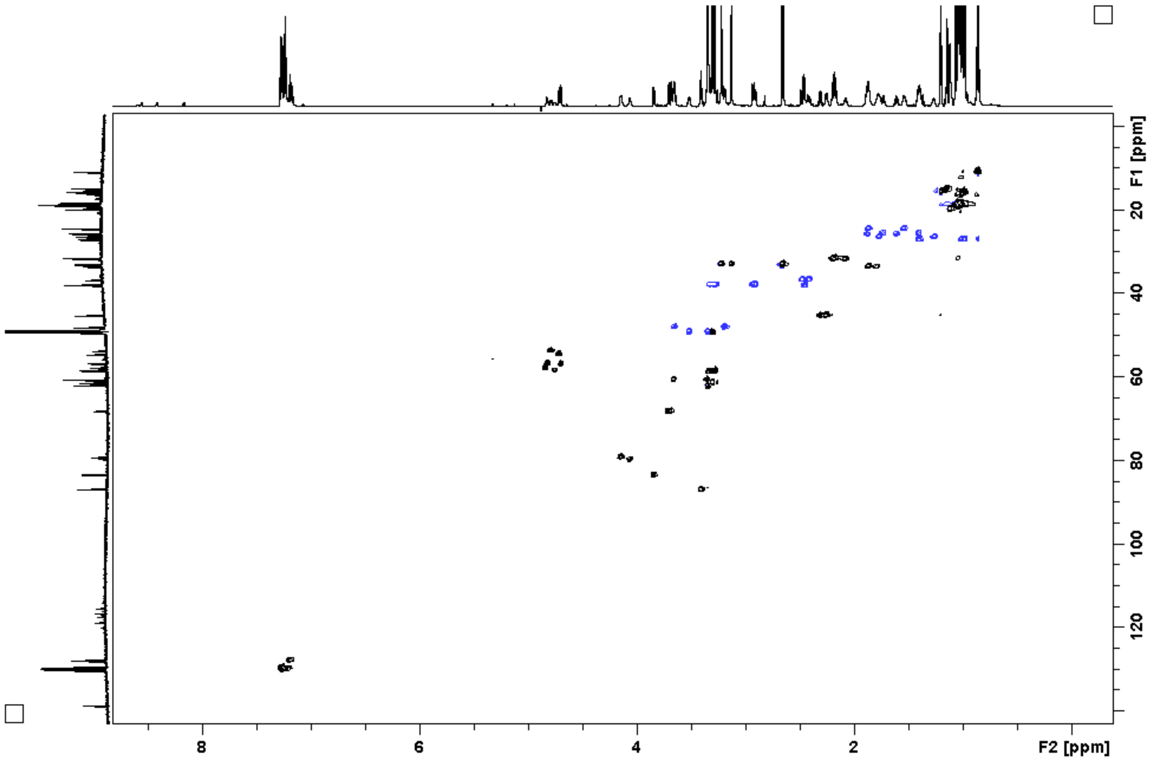

Supplementary Figure 10. EdHSQC spectrum of MMAF in CD_3OD recorded at 22 °C (850 MHz).

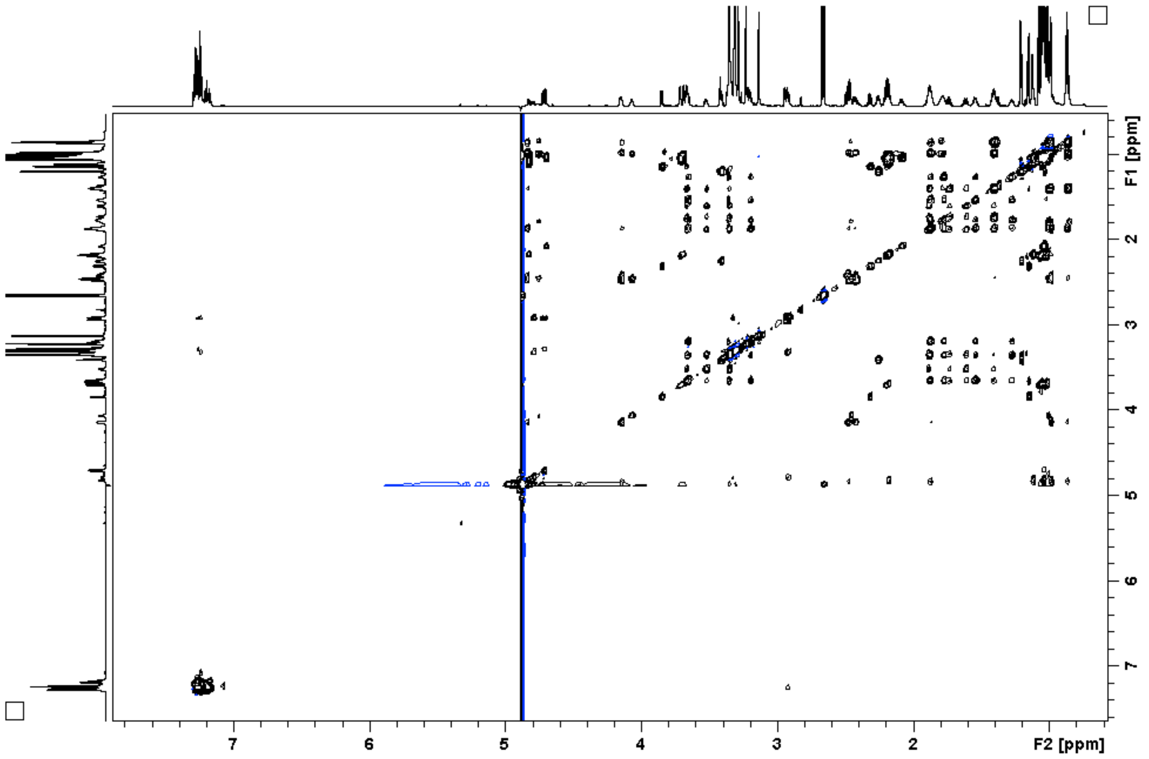

Supplementary Figure 11. 2D TOCSY spectrum of MMAF in CD_3OD recorded at 22 °C (180 ms spinlocktime) (850 MHz).

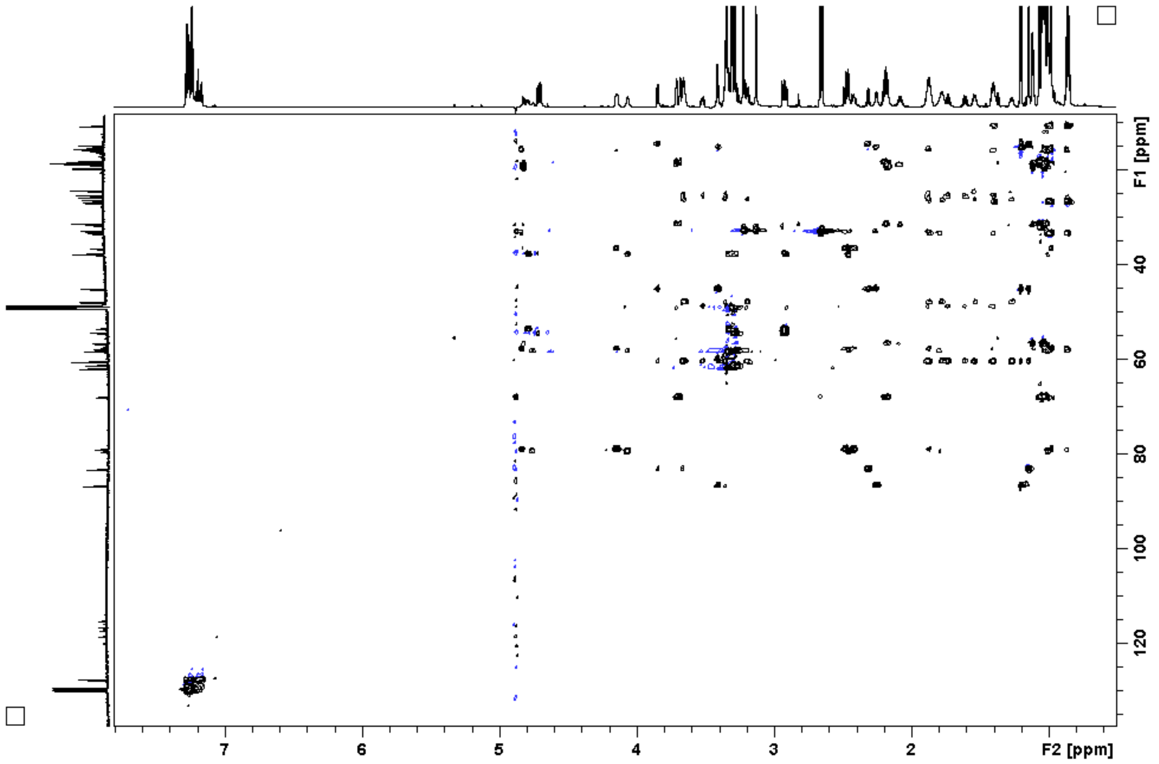

Supplementary Figure 12. 2D HSQC-TOCSY spectrum of MMAE in CD_3OD recorded at 22 °C (850 MHz).

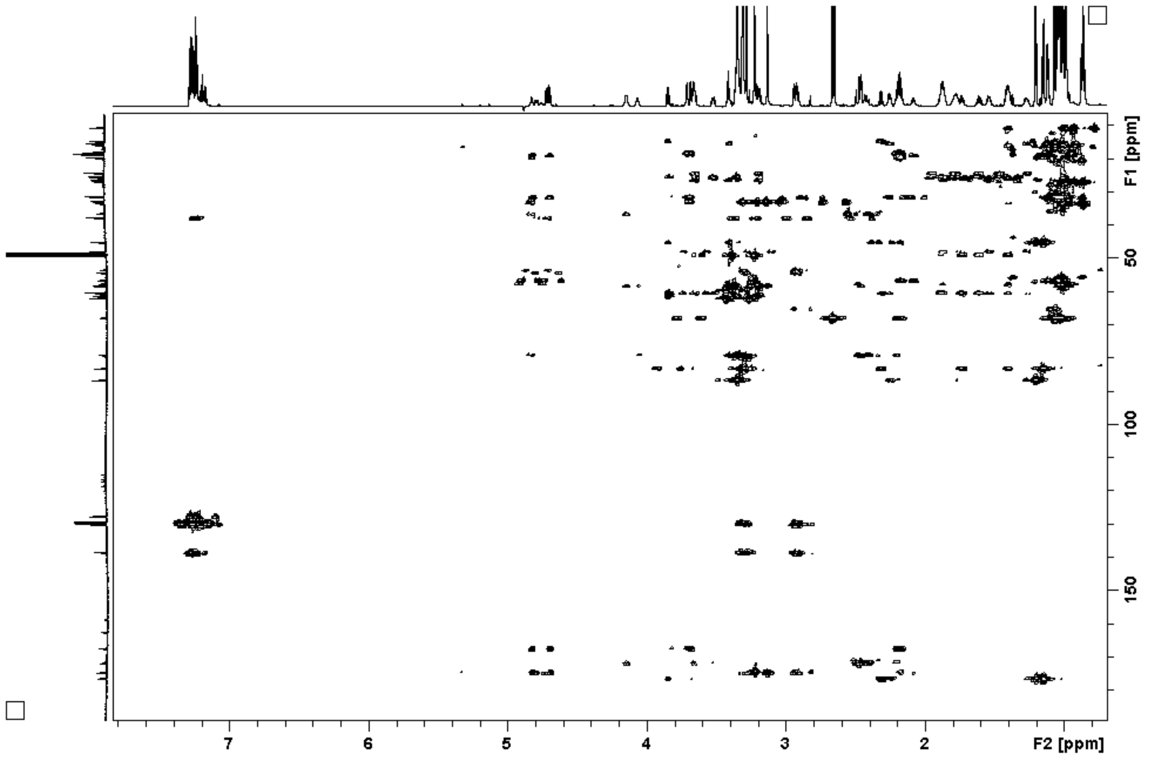

Supplementary Figure 13. HMBC spectrum of MMAF in CD_3OD recorded at 22 °C (850 MHz).

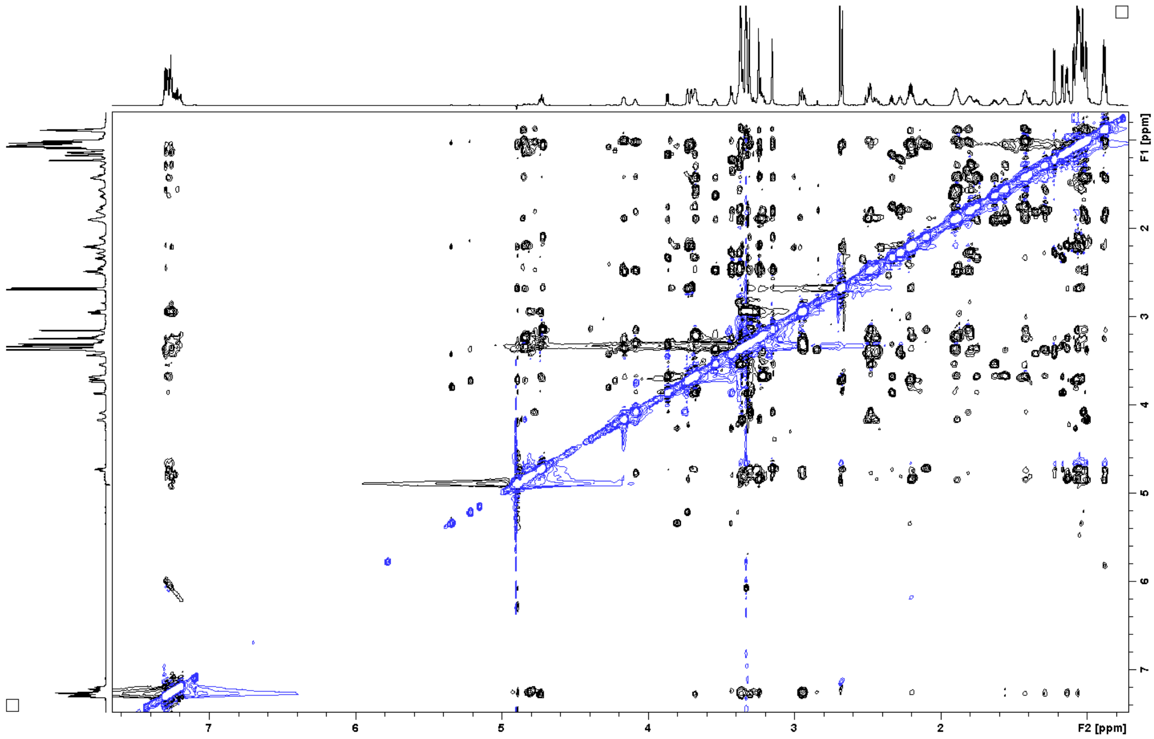

Supplementary Figure 14. 2D ROESY spectrum of MMAF in CD_3OD recorded at 22 °C (0.8 s mixing time) (850 MHz).

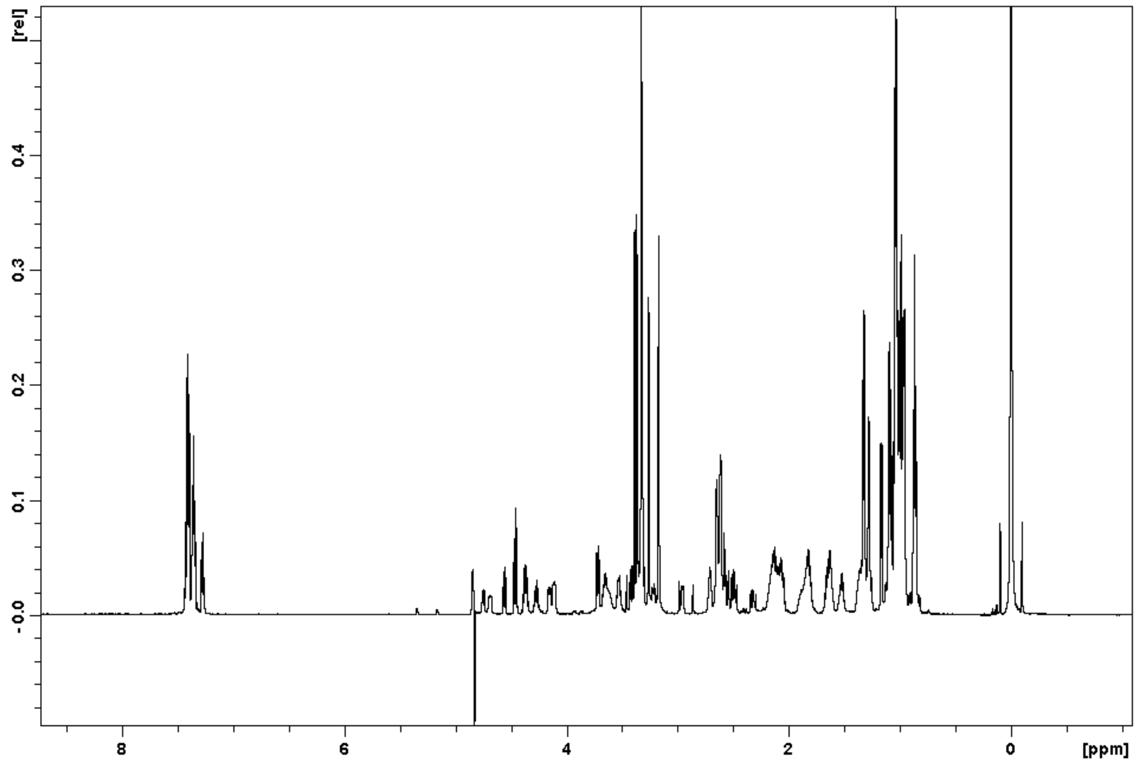

Supplementary Figure 15. ^{1}H-NMR spectrum of MMAE in D_2O recorded at 22 °C (600 MHz).

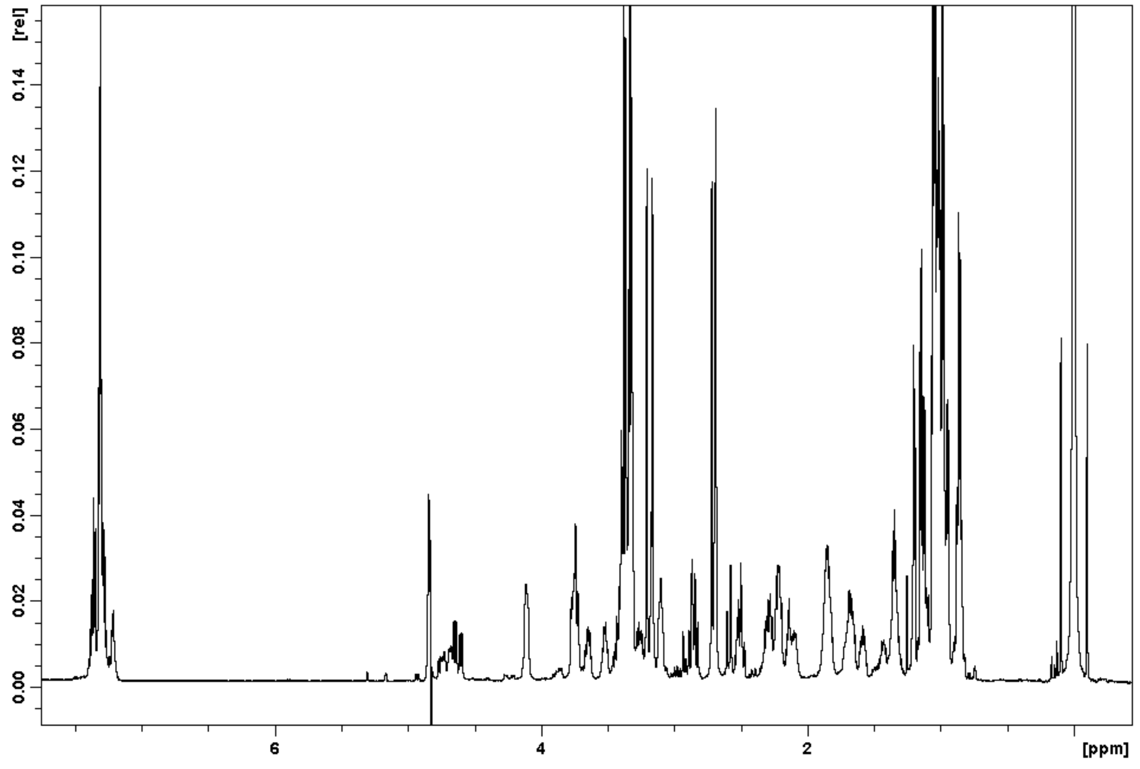

Supplementary Figure 16. ^{1}H-NMR spectrum of MMAF in D_2O recorded at 22 °C (600 MHz).

Molecular coordinates

Below we tabulate the molecular coordinates for the isomers and transition states of the studied species, in standard XYZ format (Ångström units). All geometries computed at the TPSSh-D3(BJ) density functional theory level, with the COSMO solvation model simulating a methanol environment, using the dielectric constant ε=32.6. The stable points on the potential energy surface were optimised with the def2-TZVPP basis set, and the transition states using the def2-SVP basis set.

1A: *cis*-MMAE

```
N    -0.6117916   58.5490463   21.6998763
C     0.3083166   58.5785729   22.8348631
C    -0.6318824   57.2529442   21.0395489
C    -1.8126252   57.1653532   20.0445970
C    -1.9350678   55.7596089   19.4578577
C    -1.7525471   58.2176508   18.9381413
C     0.7041157   57.0079901   20.3358654
O     1.2849313   57.8687909   19.6737053
N     1.1791018   55.7530189   20.4835501
C     2.3089915   55.2362416   19.7324678
C     1.8177829   53.9400086   19.0829806
O     1.2295990   53.1302833   19.8154087
C     3.5346179   54.9479751   20.6284784
C     4.7166768   54.4813729   19.7776776
C     3.9045064   56.1821170   21.4475379
N     2.0256234   53.7282088   17.7665407
C     2.8731674   51.5815222   16.7971374
C     0.6053241   52.5781372   16.0636447
C     1.6384834   52.4309131   17.1964921
C     3.5330830   51.9645029   15.4806211
C     4.0997627   50.7608183   14.7486155
O     3.8861978   51.6299874   17.7976609
O     4.2782086   49.6742303   15.3128351
C    -0.6178002   53.3687578   16.5427768
C    -1.5524458   53.7831456   15.4088451
C     0.2132951   51.2000811   15.5256201
C     2.7234061   54.6703463   16.9000598
C     3.5874029   50.8328230   18.9383696
C     6.0978784   50.8491079   11.8429871
C     1.0989459   53.7887726   10.9906554
C     2.5225647   53.6784131   11.5426666
C     1.6348515   50.2481702   11.8193787
C     2.7310527   54.7354222   12.6142255
N     4.4203440   50.9471031   13.4509680
C     4.2218031   52.1835492   12.6793914
C     5.2171460   52.0586705   11.5061097
C     5.1896852   49.9638956   12.6889915
C     2.7689908   52.3125073   12.1973049
O     2.4620661   51.2719428   11.2739701
O     2.0763522   54.7099912   13.6616826
O     5.6154438   58.0833484   14.2866009
C     3.2752462   58.0757140   12.7163714
N     3.6598971   55.6761499   12.3547426
C     3.9218836   56.7986713   13.2460007
C     5.4395160   56.9418172   13.4413699
C     5.8414081   55.3797350   15.3807351
C     6.8706097   54.8691492   13.2689570
C     6.4003459   54.2332086   15.9333686
C     7.4360590   53.7221224   13.8205538
C     6.0628648   55.7025382   14.0405593
C     7.1946533   53.3964425   15.1510864
H    -1.5526881   58.7485013   22.0260059
H     1.3350312   58.5080993   22.4683929
H     0.1440771   57.7558656   23.5474770
H     0.2004144   59.5256557   23.3634212
H    -0.7681173   56.4366557   21.7696154
H    -2.7050423   57.3581662   20.6528849
H    -1.9700455   54.9970767   20.2399123
```

H	-1.0876133	55.5317490	18.8076234
H	-2.8438151	55.6743859	18.8585742
H	-0.9036925	58.0363803	18.2755396
H	-1.6481983	59.2207365	19.3524267
H	-2.6672515	58.1790870	18.3417356
H	0.6347428	55.0729914	20.9952431
H	2.5734757	56.0114229	19.0186742
H	3.2481628	54.1403647	21.3104074
H	5.0465149	55.2838826	19.1105933
H	4.4637782	53.6121987	19.1670558
H	5.5618252	54.2135298	20.4150728
H	3.0733599	56.5061908	22.0751611
H	4.1787112	57.0127376	20.7916674
H	4.7559566	55.9626107	22.0953285
H	2.5272387	50.5467808	16.7160358
H	1.0499069	53.1469415	15.2402022
H	1.1530091	51.9125778	18.0221964
H	4.3635956	52.6460747	15.6797142
H	2.8488803	52.5117492	14.8363505
H	-0.2700101	54.2698678	17.0522160
H	-1.1607425	52.7816441	17.2914043
H	-2.0052748	52.9222112	14.9124079
H	-1.0059656	54.3543703	14.6533157
H	-2.3630851	54.4103756	15.7860397
H	-0.1639944	50.5614413	16.3292261
H	1.0553967	50.6865073	15.0587291
H	-0.5675675	51.2912036	14.7696577
H	3.8003455	54.5165201	16.9489575
H	2.4926919	55.6958946	17.1741935
H	2.3952883	54.5233460	15.8746005
H	3.4045762	49.7948604	18.6398906
H	2.7186852	51.2164013	19.4810155
H	4.4612717	50.8762801	19.5867676
H	6.9560196	51.1572914	12.4421496
H	6.4659948	50.3402691	10.9532712
H	0.9466718	54.7588280	10.5144366
H	0.3750360	53.6830682	11.8008859
H	0.9236147	53.0055953	10.2543035
H	3.2427298	53.8224647	10.7325201
H	0.6851670	50.6625877	12.1722466
H	2.1308463	49.7341961	12.6480184
H	1.4450422	49.5381471	11.0163069
H	4.4680957	53.0423035	13.3072131
H	4.6676371	51.8763123	10.5830606
H	5.7945059	52.9730547	11.3859835
H	5.7161703	49.3079943	13.3774069
H	4.5219958	49.3608143	12.0671565
H	2.0894786	52.2364875	13.0509516
H	6.5585722	58.1659633	14.4766983
H	2.2000299	57.9248035	12.6086030
H	3.6896951	58.3408044	11.7402944
H	3.4474070	58.9026320	13.4035355
H	4.1030700	55.6686024	11.4476868
H	3.4758653	56.5244317	14.2026462
H	5.9015881	57.1302431	12.4630369
H	5.2386251	56.0415711	15.9907506
H	7.0611126	55.1219753	12.2317037
H	6.2189176	53.9869844	16.9731900
H	8.0706479	53.0886268	13.2135838
H	7.6274490	52.5015600	15.5808742

1B: ***trans*-MMAE**

N	-0.6117916	58.5490463	21.6998763
C	0.3083166	58.5785729	22.8348631
C	-0.6318824	57.2529442	21.0395489
C	-1.8126252	57.1653532	20.0445970
C	-1.9350678	55.7596089	19.4578577
C	-1.7525471	58.2176508	18.9381413
C	0.7041157	57.0079901	20.3358654
O	1.2849313	57.8687909	19.6737053
N	1.1791018	55.7530189	20.4835501
C	2.3089915	55.2362416	19.7324678
C	1.8177829	53.9400086	19.0829806
O	1.2295990	53.1302833	19.8154087

C	3.5346179	54.9479751	20.6284784
C	4.7166768	54.4813729	19.7776776
C	3.9045064	56.1821170	21.4475379
N	2.0256234	53.7282088	17.7665407
C	2.8731674	51.5815222	16.7971374
C	0.6053241	52.5781372	16.0636447
C	1.6384834	52.4309131	17.1964921
C	3.5330830	51.9645029	15.4806211
C	4.0997627	50.7608183	14.7486155
O	3.8861978	51.6299874	17.7976609
O	4.2782086	49.6742303	15.3128351
C	-0.6178002	53.3687578	16.5427768
C	-1.5524458	53.7831456	15.4088451
C	0.2132951	51.2000811	15.5256201
C	2.7234061	54.6703463	16.9000598
C	3.5874029	50.8328230	18.9383696
C	6.0978784	50.8491079	11.8429871
C	1.0989459	53.7887726	10.9906554
C	2.5225647	53.6784131	11.5426666
C	1.6348515	50.2481702	11.8193787
C	2.7310527	54.7354222	12.6142255
N	4.4203440	50.9471031	13.4509680
C	4.2218031	52.1835492	12.6793914
C	5.2171460	52.0586705	11.5061097
C	5.1896852	49.9638956	12.6889915
C	2.7689908	52.3125073	12.1973049
O	2.4620661	51.2719428	11.2739701
O	2.0763522	54.7099912	13.6616826
O	5.6154438	58.0833484	14.2866009
C	3.2752462	58.0757140	12.7163714
N	3.6598971	55.6761499	12.3547426
C	3.9218836	56.7986713	13.2460007
C	5.4395160	56.9418172	13.4413699
C	5.8414081	55.3797350	15.3807351
C	6.8706097	54.8691492	13.2689570
C	6.4003459	54.2332086	15.9333686
C	7.4360590	53.7221224	13.8205538
C	6.0628648	55.7025382	14.0405593
C	7.1946533	53.3964425	15.1510864
H	-1.5526881	58.7485013	22.0260059
H	1.3350312	58.5080993	22.4683929
H	0.1440771	57.7558656	23.5474770
H	0.2004144	59.5256557	23.3634212
H	-0.7681173	56.4366557	21.7696154
H	-2.7050423	57.3581662	20.6528849
H	-1.9700455	54.9970767	20.2399123
H	-1.0876133	55.5317490	18.8076234
H	-2.8438151	55.6743859	18.8585742
H	-0.9036925	58.0363803	18.2755396
H	-1.6481983	59.2207365	19.3524267
H	-2.6672515	58.1790870	18.3417356
H	0.6347428	55.0729914	20.9952431
H	2.5734757	56.0114229	19.0186742
H	3.2481628	54.1403647	21.3104074
H	5.0465149	55.2838826	19.1105933
H	4.4637782	53.6121987	19.1670558
H	5.5618252	54.2135298	20.4150728
H	3.0733599	56.5061908	22.0751611
H	4.1787112	57.0127376	20.7916674
H	4.7559566	55.9626107	22.0953285
H	2.5272387	50.5467808	16.7160358
H	1.0499069	53.1469415	15.2402022
H	1.1530091	51.9125778	18.0221964
H	4.3635956	52.6460747	15.6797142
H	2.8488803	52.5117492	14.8363505
H	-0.2700101	54.2698678	17.0522160
H	-1.1607425	52.7816441	17.2914043
H	-2.0052748	52.9222112	14.9124079
H	-1.0059656	54.3543703	14.6533157
H	-2.3630851	54.4103756	15.7860397
H	-0.1639944	50.5614413	16.3292261
H	1.0553967	50.6865073	15.0587291
H	-0.5675675	51.2912036	14.7696577
H	3.8003455	54.5165201	16.9489575
H	2.4926919	55.6958946	17.1741935
H	2.3952883	54.5233460	15.8746005
H	3.4045762	49.7948604	18.6398906

H	2.7186852	51.2164013	19.4810155
H	4.4612717	50.8762801	19.5867676
H	6.9560196	51.1572914	12.4421496
H	6.4659948	50.3402691	10.9532712
H	0.9466718	54.7588280	10.5144366
H	0.3750360	53.6830682	11.8008859
H	0.9236147	53.0055953	10.2543035
H	3.2427298	53.8224647	10.7325201
H	0.6851670	50.6625877	12.1722466
H	2.1308463	49.7341961	12.6480184
H	1.4450422	49.5381471	11.0163069
H	4.4680957	53.0423035	13.3072131
H	4.6676371	51.8763123	10.5830606
H	5.7945059	52.9730547	11.3859835
H	5.7161703	49.3079943	13.3774069
H	4.5219958	49.3608143	12.0671565
H	2.0894786	52.2364875	13.0509516
H	6.5585722	58.1659633	14.4766983
H	2.2000299	57.9248035	12.6086030
H	3.6896951	58.3408044	11.7402944
H	3.4474070	58.9026320	13.4035355
H	4.1030700	55.6686024	11.4476868
H	3.4758653	56.5244317	14.2026462
H	5.9015881	57.1302431	12.4630369
H	5.2386251	56.0415711	15.9907506
H	7.0611126	55.1219753	12.2317037
H	6.2189176	53.9869844	16.9731900
H	8.0706479	53.0886268	13.2135838
H	7.6274490	52.5015600	15.5808742

1TS: MMAE 1A↔1B transition state

N	2.1753224	-5.4329896	-5.5170134
C	3.6082100	-5.4655663	-5.7770242
C	1.6019032	-4.0991504	-5.5548965
C	0.0528608	-4.1770271	-5.4806590
C	-0.5944796	-2.8069242	-5.7046083
C	-0.4462398	-4.8439773	-4.1952786
C	2.1626531	-3.2610305	-4.4004140
O	2.3070573	-3.7069584	-3.2603871
N	2.4722266	-1.9802869	-4.7284625
C	2.8286973	-0.9985816	-3.7166776
C	1.5418235	-0.2572155	-3.3227292
O	0.9305995	0.3514596	-4.2099281
C	3.8772720	0.0117200	-4.2232519
C	4.2666846	0.9667498	-3.0883311
C	5.1024263	-0.7017490	-4.8001576
N	1.1167953	-0.3270511	-2.0339471
C	0.3297260	1.7378174	-0.8815317
C	-1.1637490	-0.3898163	-0.9883066
C	-0.0656264	0.4614627	-1.6625694
C	0.7779722	1.5255970	0.5568399
C	0.5803792	2.7477911	1.4240118
O	1.3735391	2.4456638	-1.5359781
O	0.0763600	3.7742631	1.0029384
C	-1.5535283	-1.5696137	-1.8971133
C	-2.3700975	-2.6559985	-1.1978228
C	-2.3789426	0.4664222	-0.6068606
C	1.8397257	-1.0857222	-1.0132729
C	0.9432201	3.2658872	-2.6090582
C	2.1911362	2.4517093	4.7102489
C	-3.3379402	4.6115824	4.6331349
C	-2.3885289	3.4114141	4.5367764
C	-0.5114448	6.0367356	3.3067187
C	-3.0781121	2.2658789	3.7977922
N	0.9359480	2.5416614	2.7811241
C	-0.1048072	2.5809975	3.8263720
C	0.7205668	2.5939287	5.1391373
C	2.2081988	3.0392645	3.2993087
C	-1.1074469	3.7501382	3.7459237
O	-0.5120403	4.9515807	4.2200190
O	-3.2644183	2.3078434	2.5781652
O	-4.1816399	-2.3030305	3.9211445
C	-5.6515175	0.0796140	4.3797298
N	-3.4708279	1.2193576	4.5652829
C	-4.1629923	0.0579202	4.0284176

C	-3.4683151	-1.2299559	4.5190304
C	-1.5868436	-1.6671498	2.8809541
C	-1.0167456	-0.9179163	5.1076005
C	-0.2285597	-1.7139022	2.5533326
C	0.3420641	-0.9494755	4.7747572
C	-1.9933152	-1.2691777	4.1640763
C	0.7406190	-1.3436792	3.4935509
H	1.6900035	-6.0065994	-6.2091267
H	4.1471048	-4.9839591	-4.9452831
H	3.9068842	-4.9515285	-6.7161328
H	3.9464962	-6.5107421	-5.8346211
H	1.8553766	-3.5662820	-6.4996453
H	-0.2319577	-4.8241296	-6.3314663
H	-0.2627527	-2.3568059	-6.6546187
H	-0.3489475	-2.1010131	-4.8958079
H	-1.6907982	-2.9039113	-5.7407810
H	-0.2463254	-4.2106329	-3.3181282
H	0.0623675	-5.8055892	-4.0367655
H	-1.5322745	-5.0191417	-4.2538609
H	2.2251364	-1.6380188	-5.6533447
H	3.2510606	-1.5706527	-2.8840150
H	3.3898766	0.5977821	-5.0226572
H	4.7939756	0.4186309	-2.2890929
H	3.3871782	1.4549550	-2.6393909
H	4.9444588	1.7505531	-3.4604794
H	4.8283031	-1.3731057	-5.6276277
H	5.6024061	-1.3059388	-4.0243687
H	5.8297176	0.0331572	-5.1792968
H	-0.5640292	2.3842675	-0.8527274
H	-0.7589095	-0.8142028	-0.0534636
H	-0.4650054	0.8030417	-2.6274807
H	1.8389175	1.2323527	0.6118254
H	0.2215327	0.7161699	1.0581842
H	-0.6326661	-2.0218685	-2.2976846
H	-2.1066992	-1.1804523	-2.7703014
H	-3.3365279	-2.2774855	-0.8286377
H	-1.8190344	-3.0678700	-0.3357751
H	-2.5809890	-3.4889251	-1.8863345
H	-2.7547302	1.0218722	-1.4824765
H	-2.1583746	1.1874504	0.1936665
H	-3.1954556	-0.1715465	-0.2370752
H	2.7774933	-0.5901501	-0.7181658
H	2.0547672	-2.1025011	-1.3702245
H	1.2041908	-1.1650339	-0.1257821
H	0.1452652	3.9555473	-2.2783916
H	0.5848588	2.6643252	-3.4621123
H	1.8140140	3.8535101	-2.9319556
H	2.4770565	1.3900149	4.6569171
H	2.8842665	2.9649850	5.3934499
H	-4.2286644	4.3589766	5.2285105
H	-3.6672948	4.9221216	3.6293510
H	-2.8215398	5.4548602	5.1133563
H	-2.1197387	3.0837805	5.5554182
H	-1.4961038	6.1644882	2.8247370
H	0.2459358	5.8965009	2.5173894
H	-0.2806520	6.9441218	3.8835232
H	-0.6872346	1.6472399	3.7391773
H	0.5689418	3.5592367	5.6411322
H	0.4039641	1.7946093	5.8227023
H	3.0470079	2.6724301	2.6862385
H	2.2569543	4.1472523	3.3230609
H	-1.4130673	3.8788992	2.6987790
H	-3.7777394	-3.1313351	4.2272426
H	-6.1206880	0.9873013	3.9714746
H	-5.7925697	0.0745472	5.4730162
H	-6.1528187	-0.8027307	3.9593829
H	-3.3297928	1.2804971	5.5699819
H	-4.0384358	0.1195377	2.9371876
H	-3.5632543	-1.2684243	5.6226724
H	-2.3442327	-1.9655627	2.1526466
H	-1.3225613	-0.6228425	6.1151894
H	0.0790009	-2.0611000	1.5642045
H	1.0914036	-0.6760350	5.5207350
H	1.8007912	-1.3752842	3.2325186

2A: *cis*-MMAF

N	-0.6378670	58.5345074	21.6661318
C	0.2754871	58.5640305	22.8064634
C	-0.6498206	57.2405591	21.0012410
C	-1.8218483	57.1534678	19.9962829
C	-1.9271642	55.7523102	19.3957641
C	-1.7619081	58.2162864	18.8999594
C	0.6932218	57.0007070	20.3088316
O	1.2713272	57.8608811	19.6435293
N	1.1798690	55.7519338	20.4728450
C	2.3198971	55.2389596	19.7338322
C	1.8433122	53.9406977	19.0783180
O	1.2625258	53.1215319	19.8060039
C	3.5363727	54.9552211	20.6433786
C	4.7332712	54.5102997	19.8023323
C	3.8824045	56.1848631	21.4792931
N	2.0554619	53.7383183	17.7610140
C	2.9241154	51.6028132	16.7814092
C	0.6480219	52.5813051	16.0510379
C	1.6812950	52.4401202	17.1841640
C	3.5805471	51.9916361	15.4627900
C	4.1239479	50.7841727	14.7175157
O	3.9363559	51.6573982	17.7819591
O	4.3259591	49.7039068	15.2850707
C	-0.5857946	53.3566478	16.5273869
C	-1.5080779	53.7792942	15.3863146
C	0.2709053	51.2017624	15.5064398
C	2.7398021	54.6976903	16.9013410
C	3.6443060	50.8523635	18.9191321
C	6.0351318	50.8401078	11.7602006
C	1.0474769	53.8418007	11.0219835
C	2.4823878	53.7103258	11.5392880
C	1.5787466	50.2789737	11.8167397
C	2.7284371	54.7564374	12.6116948
N	4.3927610	50.9558216	13.4052240
C	4.1883293	52.1916006	12.6350365
C	5.1602218	52.0594569	11.4427835
C	5.1374518	49.9649254	12.6278650
C	2.7275895	52.3371175	12.1823051
O	2.3885835	51.3095693	11.2570631
O	2.0949962	54.7487167	13.6674391
H	5.6998282	57.7753451	14.0848049
C	3.4101309	58.0389655	12.7206863
N	3.6881273	55.6736757	12.3452434
C	4.0192333	56.7506702	13.2661805
C	5.5314652	56.8923958	13.4646092
C	5.9543363	55.4589528	15.4912625
C	6.8551899	54.7407315	13.3840609
C	6.4723684	54.3233859	16.1042724
C	7.3824226	53.6058023	13.9955829
C	6.1299503	55.6765829	14.1223462
C	7.1836879	53.3887017	15.3549441
H	-1.5813608	58.7305462	21.9867268
H	1.3042624	58.4931071	22.4460114
H	0.1070565	57.7417514	23.5185859
H	0.1648969	59.5114220	23.3339038
H	-0.7905909	56.4215894	21.7274874
H	-2.7205330	57.3334141	20.5992025
H	-1.9623241	54.9820784	20.1701881
H	-1.0717928	55.5384757	18.7511672
H	-2.8294480	55.6645228	18.7871901
H	-0.9098400	58.0445816	18.2389538
H	-1.6632504	59.2158669	19.3239506
H	-2.6742649	58.1793952	18.2998597
H	0.6342262	55.0705842	20.9813836
H	2.5891320	56.0153205	19.0230358
H	3.2499063	54.1377237	21.3133220
H	5.0705858	55.3287218	19.1587830
H	4.4925748	53.6540579	19.1686617
H	5.5688132	54.2299912	20.4470100
H	3.0429603	56.4884696	22.1059470
H	4.1474345	57.0276095	20.8351669
H	4.7336508	55.9705782	22.1290361

H	2.5863576	50.5655800	16.6990010
H	1.0884891	53.1585869	15.2313327
H	1.2010745	51.9132245	18.0074574
H	4.4226523	52.6587747	15.6648780
H	2.8993652	52.5536336	14.8282994
H	-0.2510370	54.2530263	17.0533219
H	-1.1336679	52.7555673	17.2611134
H	-1.9507025	52.9217750	14.8750332
H	-0.9536830	54.3605703	14.6442174
H	-2.3260786	54.3994529	15.7591808
H	-0.1033538	50.5572001	16.3066647
H	1.1189577	50.6968978	15.0410471
H	-0.5084974	51.2882229	14.7484050
H	3.8208322	54.5798158	16.9671916
H	2.4700019	55.7170565	17.1638839
H	2.4319160	54.5345450	15.8725374
H	3.4653605	49.8152251	18.6155108
H	2.7751447	51.2288869	19.4659083
H	4.5196655	50.8959592	19.5654845
H	6.9110392	51.1385103	12.3379295
H	6.3758962	50.3269047	10.8621666
H	0.8923712	54.8195139	10.5629777
H	0.3410689	53.7298051	11.8465812
H	0.8501926	53.0704620	10.2790390
H	3.1859792	53.8504131	10.7142461
H	0.6395621	50.6892786	12.2008065
H	2.0990961	49.7581592	12.6256518
H	1.3665724	49.5767685	11.0125061
H	4.4549977	53.0472855	13.2578519
H	4.5916209	51.8850742	10.5297952
H	5.7451692	52.9679382	11.3128354
H	5.6727709	49.3055248	13.3058947
H	4.4510444	49.3666857	12.0219483
H	2.0645017	52.2628947	13.0494124
H	4.1388463	55.6584394	11.4421564
H	3.5208808	56.5028166	14.2020759
H	6.0034937	57.0791610	12.4983027
H	5.4094522	56.1899835	16.0789951
H	7.0154276	54.9060972	12.3246026
H	6.3231086	54.1628374	17.1653399
H	7.9526922	52.8943955	13.4115171
H	7.5827582	52.5011967	15.8301718
O	3.9838506	58.8215449	11.9941687
O	2.1370800	58.1848452	13.1115371
H	1.7755870	58.9882418	12.6988022

2B: *trans*-MMAF

N	-0.2406557	58.6041143	20.1379034
C	0.9955919	59.2735267	20.5378677
C	-0.1284006	57.1545512	20.1914085
C	-1.5224353	56.4969032	20.0535083
C	-1.4570242	54.9943345	20.3279970
C	-2.1987525	56.7870619	18.7139347
C	0.8219094	56.6678962	19.0961421
O	0.8171040	57.1205348	17.9506729
N	1.6602767	55.6818311	19.4874000
C	2.4745923	54.9296839	18.5468941
C	1.7412484	53.6126132	18.2686524
O	1.4922013	52.8751589	19.2300620
C	3.8782038	54.6427705	19.1068446
C	4.6984409	53.8569907	18.0827664
C	4.5792473	55.9423985	19.4973209
N	1.3818677	53.3273884	16.9971669
C	1.7818256	51.0296797	16.1417219
C	-0.5557635	52.1396306	15.9383669
C	0.7580325	52.0206952	16.7318990
C	2.2577450	51.3456084	14.7347155
C	3.1032164	50.1971882	14.2089780
O	2.9594792	50.9896409	16.9457660
O	2.8320037	49.0243755	14.4848780
C	-1.5210615	53.0856283	16.6677672
C	-2.7647766	53.4521459	15.8635758
C	-1.1728496	50.7539928	15.7287917
C	1.6255928	54.2607217	15.8957735
C	2.8592764	50.1208236	18.0654008

C	5.1941170	51.5387951	11.6102859
C	7.6415451	47.1418911	14.6696585
C	6.8195929	47.7505973	13.5322529
C	6.2006541	50.0964851	16.0581042
C	6.0045992	46.6628151	12.8565838
N	4.1633452	50.5245217	13.4445452
C	5.0678430	49.4904606	12.9180038
C	5.9533530	50.2466492	11.9144134
C	4.4854318	51.8567361	12.9228385
C	5.8661850	48.8396316	14.0522665
O	6.6340637	49.8407482	14.7230480
O	5.2158587	45.9536954	13.4821390
H	4.2032948	45.3083351	9.0721984
C	6.4505969	44.4195609	10.2632145
N	6.2024825	46.5287775	11.5232810
C	5.5177990	45.5253705	10.7398005
C	4.7247773	46.1258185	9.5736892
C	2.8182082	46.9297446	11.0366682
C	3.7472089	48.4416873	9.4203321
C	1.9198655	47.9109010	11.4406777
C	2.8373894	49.4210931	9.8107446
C	3.7458644	47.1825684	10.0220843
C	1.9235035	49.1599898	10.8259129
H	-0.9894173	58.8821475	20.7650686
H	1.7678800	59.0818656	19.7895153
H	1.3747209	58.9348262	21.5141500
H	0.8257311	60.3493274	20.5836886
H	0.2895315	56.8228016	21.1575091
H	-2.1248910	56.9576273	20.8461553
H	-1.0043028	54.7859308	21.3008939
H	-0.8704288	54.4742568	19.5673847
H	-2.4610756	54.5647181	20.3213093
H	-1.6722094	56.2943739	17.8945359
H	-2.2166870	57.8572710	18.5060174
H	-3.2263090	56.4161608	18.7306313
H	1.5421533	55.2903308	20.4106380
H	2.5605843	55.5533810	17.6616330
H	3.7466813	54.0212551	19.9989518
H	4.8728065	54.4614081	17.1871770
H	4.1983451	52.9354150	17.7791533
H	5.6730440	53.5943383	18.4999147
H	4.0043457	56.5023265	20.2367437
H	4.7195478	56.5825446	18.6211881
H	5.5630666	55.7282663	19.9203377
H	1.3323069	50.0332151	16.1369717
H	-0.3512374	52.5643912	14.9487964
H	0.5033529	51.6468003	17.7243231
H	2.8315845	52.2684549	14.7444690
H	1.4077902	51.4876532	14.0611012
H	-0.9860476	54.0040524	16.9199006
H	-1.8087270	52.6283238	17.6206216
H	-3.3899487	52.5826151	15.6501504
H	-2.4882157	53.9091037	14.9093528
H	-3.3768743	54.1708668	16.4128580
H	-1.3075933	50.2430541	16.6865269
H	-0.5505711	50.1217219	15.0934867
H	-2.1485039	50.8371147	15.2487749
H	2.6869671	54.3263637	15.6499290
H	1.2511045	55.2536474	16.1432053
H	1.0936059	53.9065903	15.0181537
H	2.5911567	49.1103994	17.7396853
H	2.1272233	50.4879795	18.7901063
H	3.8431975	50.1019468	18.5325035
H	4.4489359	51.3620499	10.8343370
H	5.8457980	52.3489448	11.2858897
H	8.3456556	46.4026066	14.2836537
H	6.9840787	46.6510973	15.3887335
H	8.2022921	47.9248188	15.1787356
H	7.4956055	48.2046311	12.8019295
H	6.2894996	49.1954377	16.6745858
H	5.1671820	50.4481837	16.0831260
H	6.8574372	50.8694727	16.4565650
H	4.4620563	48.7254321	12.4273319
H	6.9138091	50.4776950	12.3774498
H	6.1346164	49.6534115	11.0194781
H	3.5825215	52.4435901	12.7713434
H	5.1433019	52.3883933	13.6177215

H	5.1622702	48.3848227	14.7519043
H	6.9053086	47.0999431	11.0795384
H	4.8326633	45.0428980	11.4434549
H	5.4169514	46.5548889	8.8457889
H	2.8033085	45.9637021	11.5279833
H	4.4659194	48.6555346	8.6367454
H	1.2257639	47.7053963	12.2455612
H	2.8486130	50.3896732	9.3253979
H	1.2248351	49.9256144	11.1412793
O	6.3161623	43.8154763	9.2205624
O	7.4185289	44.1500204	11.1503673
H	7.9511045	43.4133008	10.8052650

2TS: MMAF 2A↔2B transition state

N	2.3361999	-5.6625562	-5.0783919
C	3.7803495	-5.7048197	-5.2645908
C	1.7663007	-4.3331636	-5.2106118
C	0.2153965	-4.4104044	-5.2110762
C	-0.4194525	-3.0549674	-5.5371327
C	-0.3481711	-5.0120208	-3.9200950
C	2.2678743	-3.4389812	-4.0711241
O	2.3571860	-3.8303839	-2.9057745
N	2.5913412	-2.1740594	-4.4433658
C	2.9003729	-1.1453475	-3.4633829
C	1.5983381	-0.3856201	-3.1662834
O	1.0342285	0.1854559	-4.1080936
C	3.9744192	-0.1599605	-3.9658265
C	4.3068504	0.8509546	-2.8615759
C	5.2262399	-0.8997891	-4.4436745
N	1.1116796	-0.3968759	-1.8977522
C	0.2845248	1.7155889	-0.8707576
C	-1.2224281	-0.4067488	-0.9758297
C	-0.0817679	0.4129318	-1.6186781
C	0.6858950	1.5613880	0.5882290
C	0.4838900	2.8271176	1.3889492
O	1.3526378	2.3986478	-1.5144499
O	-0.0025275	3.8358844	0.9087664
C	-1.5849672	-1.5941343	-1.8872438
C	-2.4626152	-2.6579016	-1.2288200
C	-2.4416653	0.4741661	-0.6718489
C	1.7738283	-1.1200727	-0.8120260
C	0.9608612	3.1741535	-2.6339022
C	2.0711378	2.6994877	4.6985059
C	-3.5489277	4.6968391	4.4109788
C	-2.5515883	3.5319190	4.4013873
C	-0.5936551	6.1574139	3.2003589
C	-3.1652482	2.3407260	3.6733968
N	0.8285233	2.6934567	2.7581680
C	-0.2211743	2.7501363	3.7907364
C	0.5919396	2.8146597	5.1093992
C	2.0892225	3.2297280	3.2647907
C	-1.2479500	3.8981143	3.6633207
O	-0.7187403	5.1203719	4.1603345
O	-3.3143823	2.3322989	2.4496492
H	-4.1054439	-2.0242257	4.0897612
C	-5.6745391	0.0755352	4.1738676
N	-3.5352456	1.2966955	4.4601493
C	-4.1646503	0.1101450	3.9307500
C	-3.4968338	-1.1804893	4.4425363
C	-1.7823922	-1.9963744	2.7699105
C	-1.0051592	-0.7749691	4.7022661
C	-0.4631808	-2.1291572	2.3233457
C	0.3129922	-0.8887780	4.2477729
C	-2.0707481	-1.3236472	3.9688283
C	0.5891853	-1.5674379	3.0560524
H	1.8861210	-6.2701222	-5.7653396
H	4.2774026	-5.1838562	-4.4305098
H	4.1256795	-5.2347529	-6.2106817
H	4.1204171	-6.7509780	-5.2562413
H	2.0677404	-3.8463556	-6.1660474
H	-0.0265776	-5.1006455	-6.0410224
H	-0.0435616	-2.6556836	-6.4933333
H	-0.2109767	-2.3065844	-4.7565091
H	-1.5130477	-3.1553079	-5.6187393
H	-0.1887246	-4.3359481	-3.0667509
H	0.1483763	-5.9652177	-3.6890872

H	-1.4307871	-5.1882004	-4.0224606
H	2.3960123	-1.8779727	-5.3961206
H	3.2811095	-1.6770746	-2.5850241
H	3.5284299	0.3850158	-4.8167737
H	4.7967885	0.3448586	-2.0124762
H	3.4058059	1.3563119	-2.4792280
H	4.9996034	1.6190048	-3.2390087
H	4.9934837	-1.6128103	-5.2487227
H	5.6861109	-1.4627795	-3.6140200
H	5.9722781	-0.1839293	-4.8228690
H	-0.6087744	2.3627896	-0.8974742
H	-0.8699070	-0.8179959	-0.0137510
H	-0.4272808	0.7203417	-2.6152657
H	1.7404401	1.2580787	0.6890458
H	0.1044047	0.7810471	1.1081435
H	-0.6521827	-2.0687244	-2.2305815
H	-2.0790956	-1.2052044	-2.7953680
H	-3.4298209	-2.2507097	-0.8939150
H	-1.9612363	-3.0956592	-0.3503632
H	-2.6731072	-3.4777569	-1.9329283
H	-2.7603720	1.0250598	-1.5728281
H	-2.2516945	1.2009873	0.1312420
H	-3.2877339	-0.1461449	-0.3403917
H	2.7003434	-0.6207179	-0.4884353
H	1.9992232	-2.1510605	-1.1187725
H	1.0926766	-1.1593749	0.0438808
H	0.1849537	3.9080994	-2.3489548
H	0.5899399	2.5414509	-3.4584625
H	1.8548905	3.7120899	-2.9789740
H	2.3922431	1.6466978	4.6930796
H	2.7406951	3.2609474	5.3669461
H	-4.4605467	4.4272291	4.9655824
H	-3.8339486	4.9641561	3.3815775
H	-3.0877208	5.5722784	4.8893197
H	-2.3196016	3.2469834	5.4417187
H	-1.5711942	6.4180709	2.7557416
H	0.0946151	5.8727715	2.3880113
H	-0.2002250	7.0366596	3.7299536
H	-0.7875054	1.8050650	3.7252188
H	0.4084147	3.7858066	5.5884721
H	0.2911128	2.0220441	5.8090242
H	2.9376425	2.8506983	2.6728262
H	2.1246465	4.3385315	3.2432855
H	-1.5113917	4.0143484	2.6025298
H	-3.4345355	1.3849665	5.4670484
H	-4.0470237	0.1793436	2.8353140
H	-3.5375771	-1.1844754	5.5438309
H	-2.5988906	-2.4355210	2.1901106
H	-1.2097691	-0.2592420	5.6439236
H	-0.2544647	-2.6882283	1.4083562
H	1.1275509	-0.4543431	4.8308808
H	1.6197116	-1.6682371	2.7075278
O	-6.3405902	-0.9384463	4.1207692
O	-6.1888331	1.2856643	4.4110175
H	-7.1566842	1.1875525	4.5149005

2C: internally hydrogen bonded MMAF

N	0.5214915	-4.6214203	0.3975345
C	0.8564307	-4.5996631	1.8204589
C	1.4920284	-3.9205205	-0.4350293
C	1.0915586	-3.9532322	-1.9209015
C	0.7561541	-5.3662367	-2.3912174
C	2.1936173	-3.3296772	-2.7796630
C	1.6257646	-2.4595646	-0.0020987
O	0.6519989	-1.6945157	-0.0294624
N	2.8354464	-2.0693288	0.4388346
C	3.1187652	-0.6847671	0.7730734
C	4.0086420	-0.1016576	-0.3313202
O	4.8961424	-0.8118289	-0.8233156
C	3.7518221	-0.5222340	2.1739349
C	2.7288081	-0.9143395	3.2412777
C	5.0630003	-1.2891917	2.3435633
N	3.7857700	1.1716790	-0.7322691
C	1.3715150	1.6057585	-0.9324112

C	2.9650508	3.4660677	0.0149358
C	2.6692756	1.9673569	-0.1875789
C	0.1176759	2.3124280	-0.4267418
C	-0.2560205	1.9375068	0.9922289
O	1.4628976	1.8424417	-2.3460076
O	0.5766689	1.5306148	1.8158184
C	3.0110952	4.3751606	-1.2267610
C	2.6668708	5.8235216	-0.8795137
C	4.1983190	3.6350449	0.9061993
C	4.5662477	1.6843564	-1.8557144
C	1.6002716	0.6493892	-3.1069990
C	-3.8007490	2.7748239	1.3885248
C	-1.8707131	-2.0152600	2.8652774
C	-1.8357036	-0.6306182	2.1943839
C	-2.4063688	0.8766078	5.4864569
C	-2.7458979	-0.7334628	0.9802716
N	-1.5577145	2.0682232	1.3184441
C	-1.9884714	1.9202071	2.7153066
C	-3.1820846	2.8778926	2.7854701
C	-2.5840390	2.6980663	0.4692158
C	-2.3225681	0.4620959	3.1466522
O	-1.7388058	0.2104531	4.4255475
O	-3.9640194	-0.5420994	1.0486223
H	-3.0573015	-1.4712295	-3.4095437
C	-2.8335131	-3.1450173	-1.3658052
N	-2.1388102	-1.1477167	-0.1569297
C	-2.9148873	-1.6255960	-1.2757992
C	-2.5668165	-0.9271604	-2.6009311
C	-2.1084936	1.5057983	-3.0774890
C	-4.2972239	0.8996364	-2.3053924
C	-2.5007542	2.8396060	-3.1588967
C	-4.6946296	2.2296433	-2.3879574
C	-2.9954691	0.5194242	-2.6461316
C	-3.7966123	3.2066289	-2.8125797
H	-0.3939475	-4.2012083	0.2517688
H	0.9413502	-3.5890459	2.2446272
H	1.8096258	-5.1112771	1.9737812
H	0.0853618	-5.1356652	2.3748713
H	2.4619902	-4.4153658	-0.3071624
H	0.1941312	-3.3316235	-2.0126594
H	1.6241303	-6.0244800	-2.2889479
H	0.4664657	-5.3491630	-3.4448898
H	-0.0620604	-5.7921118	-1.8117007
H	2.4473742	-2.3216020	-2.4446375
H	1.8775490	-3.2649504	-3.8226212
H	3.1043354	-3.9340239	-2.7370731
H	3.6176371	-2.6934067	0.3059044
H	2.1441870	-0.2083369	0.8018980
H	3.9621870	0.5481439	2.2753414
H	1.8176102	-0.3227210	3.1423019
H	3.1422928	-0.7603550	4.2404927
H	2.4649104	-1.9707425	3.1460179
H	4.8942825	-2.3691471	2.2915098
H	5.4933085	-1.0710543	3.3229865
H	5.7914332	-1.0211767	1.5776519
H	1.2170434	0.5325102	-0.7834048
H	2.1065339	3.8009549	0.6094443
H	2.5021949	1.6166304	0.8262581
H	-0.6991984	2.0478787	-1.0971105
H	0.2217695	3.4001650	-0.4914241
H	2.3101616	4.0079513	-1.9776198
H	4.0041500	4.3394860	-1.6820786
H	2.7146694	6.4612777	-1.7650858
H	3.3537780	6.2358937	-0.1363346
H	1.6546450	5.8918677	-0.4702905
H	4.3454973	4.6836884	1.1692310
H	5.1042337	3.2853498	0.4070504
H	4.0868508	3.0695094	1.8347276
H	5.0610703	0.8434432	-2.3328251
H	5.3208184	2.3969753	-1.5189427
H	3.8979855	2.1766081	-2.5573300
H	2.5671348	0.1683048	-2.9320581

H	1.5251944	0.9304686	-4.1560928
H	0.8069766	-0.0654082	-2.8632826
H	-4.4448033	3.6174138	1.1404697
H	-4.3773016	1.8534622	1.3008836
H	-1.1705524	-2.0485747	3.6970284
H	-1.5968773	-2.7809751	2.1390959
H	-2.8727346	-2.2350259	3.2398430
H	-0.8146032	-0.4244082	1.8727816
H	-1.9768333	0.5020935	6.4148128
H	-3.4805272	0.6576945	5.4655224
H	-2.2660688	1.9626976	5.4439581
H	-1.1582962	2.2334011	3.3483580
H	-3.8772857	2.6122407	3.5810268
H	-2.8234057	3.8938692	2.9682111
H	-2.2513051	3.6910139	0.1567879
H	-2.7694887	2.0960155	-0.4185057
H	-3.4107292	0.3571576	3.2385289
H	-1.1498836	-1.4024797	-0.1134513
H	-3.9591199	-1.4175870	-1.0271453
H	-1.4910408	-1.0016809	-2.7764459
H	-1.0961977	1.2315049	-3.3469924
H	-5.0097480	0.1556959	-1.9687049
H	-1.7875257	3.5895729	-3.4795185
H	-5.7055813	2.5056115	-2.1125480
H	-4.1039104	4.2440317	-2.8648184
O	-3.4040146	-3.6122595	-2.4868628
O	-2.3565469	-3.8700474	-0.5200312
H	-3.3754086	-4.5838789	-2.4622681
